# Supplementary material for: An Integrative Review Considering the Impact of Storytelling and Sharing Interventions in Stroke
Source: Behav Sci (Basel). 2021 Jun 16;11(6):88. doi: 10.3390/bs11060088 (PMC8234102; doi:10.3390/bs11060088)
Supplement: Supplementary file 1 [file behavsci-11-00088-s001.zip › behavsci-1218777-supplementary.pdf]

Supplementary File

Table S1: Enhancing transparency in reporting the synthesis of qualitative research: the ENTREQ statement (Tong et al., 2012)

No	Item	Guide and description	Page number
**1**	Aim	State the research question the synthesis addresses.	1-2
**2**	Synthesis methodology	Identify the synthesis methodology or theoretical framework which underpins the synthesis and describe the rationale for choice of methodology *(e.g. meta-ethnography, thematic synthesis, critical interpretive synthesis, grounded theory synthesis, realist synthesis, meta-aggregation, meta-study, framework synthesis).*	4-5
**3**	Approach to searching	Indicate whether the search was pre-planned (*comprehensive search strategies to seek all available studies*) or iterative (*to seek all available concepts until they theoretical saturation is achieved*).	3-4
**4**	Inclusion criteria	Specify the inclusion/exclusion criteria *(e.g. in terms of population, language, year limits, type of publication, study type).*	2-3
**5**	Data sources	Describe the information sources used (e.g. *electronic databases (MEDLINE, EMBASE, CINAHL, psycINFO, Econlit), grey literature databases (digital thesis, policy reports), relevant organisational websites, experts, information specialists, generic web*	3-4

No	Item	Guide and description	Page number
		searches (Google Scholar) hand searching, reference lists) and when the searches conducted; provide the rationale for using the data sources.	
**6**	Electronic Search strategy	Describe the literature search *(e.g. provide electronic search strategies with population terms, clinical or health topic terms, experiential or social phenomena related terms, filters for qualitative research, and search limits).*	**3-4**
**7**	Study screening methods	Describe the process of study screening and sifting *(e.g. title, abstract and full text review, number of independent reviewers who screened studies).*	**3-4**
**8**	Study characteristics	Present the characteristics of the included studies *(e.g. year of publication, country, population, number of participants, data collection, methodology, analysis, research questions).*	In supplementary file Table S2
**9**	Study selection results	Identify the number of studies screened and provide reasons for study exclusion *(e,g, for comprehensive searching, provide numbers of studies screened and reasons for exclusion indicated in a figure/flowchart; for iterative searching describe reasons for study exclusion and inclusion based on modifications t the research question and/or contribution to theory development).*	5
**10**	Rationale for appraisal	Describe the rationale and approach used to appraise the included studies or selected findings *(e.g. assessment of conduct (validity and robustness), assessment of reporting (transparency), assessment of content and utility of the findings).*	6
**11**	Appraisal items	State the tools, frameworks and criteria used to appraise the studies or selected findings *(e.g. Existing tools: CASP, QARI, COREQ, Mays and Pope*[25]*; reviewer*	6

No	Item	Guide and description	Page number
		developed tools; describe the domains assessed: research team, study design, data analysis and interpretations, reporting).	
**12**	Appraisal process	Indicate whether the appraisal was conducted independently by more than one reviewer and if consensus was required.	6
**13**	Appraisal results	Present results of the quality assessment and indicate which articles, if any, were weighted/excluded based on the assessment and give the rationale.	Page 6 & Supplementary files S3 and S4
**14**	Data extraction	Indicate which sections of the primary studies were analysed and how were the data extracted from the primary studies? *(e.g. all text under the headings "results /conclusions" were extracted electronically and entered into a computer software).*	4 and 5
**15**	Software	State the computer software used, if any.	n/a
**16**	Number of reviewers	Identify who was involved in coding and analysis.	. R.A and A.S ,
**17**	Coding	Describe the process for coding of data *(e.g. line by line coding to search for concepts).*	Supplementary file S5
**18**	Study comparison	Describe how were comparisons made within and across studies *(e.g. subsequent studies were coded into pre-existing concepts, and new concepts were created when deemed necessary).*	S7

No	Item	Guide and description	Page number
**19**	Derivation of themes	Explain whether the process of deriving the themes or constructs was inductive or deductive.	Inductive process
**20**	Quotations	Provide quotations from the primary studies to illustrate themes/constructs and identify whether the quotations were participant quotations of the author's interpretation.	S8 and S9
**21**	Synthesis output	Present rich, compelling and useful results that go beyond a summary of the primary studies (e.g. *new interpretation, models of evidence, conceptual models, analytical framework, development of a new theory or construct).*	Page 6-10

S2: The demographic and design-related information on included studies

Study	Methodology, aim & Eligibility	Participants, Sampling and Setting	Key intervention details in line with TIDieR checklist(Hoffmann et al., 2014)	Data collection methods, aim and focus and analysis
**Gurr (2009)**	Design:<br>Mixed methodology, including depression outcome measures and qualitative themes<br><br>Aim:<br>To explain the application and assessment of psychosocial group sessions for stroke patients in acute stage of rehabilitation<br><br>Eligibility : Stroke patients admitted to stroke rehabilitation unit with minimum length of stay 2 weeks, medically stable, could give consent, physically able to sit in a chair or wheelchair, could communicate with or without the assistance of communication aids and cognitively able to sustain concentration throughout the sessions.<br><br>Country :UK	Sample size: 80 stroke survivors (41 males and 39 females)<br><br>Age: Not reported<br><br>Living condition: Not reported<br><br>Material Status: Not reported<br><br>Time since stroke: Acute phase of rehabilitation<br><br>Type/location of stroke : Not reported<br><br>co-morbidity/severity of stroke, : Not reported<br><br>Recruitment: sampling strategies not identified	The intervention: "Share your story group" (personal narrative group), no control group<br><br>Developmental rationale: Developed and assessed by working party of 2 speech/ language therapists and 2 trainee clinical psychologists aiming to:<br><br>(1) Create a space where thoughts and feelings related to impact of their stroke and rehabilitation progress could be shared.<br>(2) Create a safe environment for common emotional support.<br>(3) Improve social interaction and peer support<br>(4) Enhance rehabilitation opportunities<br><br>Content:<br><br>Two sessions:<br><br>First session: Introduction and ground rules. Participants invited to share their experiences of diagnosis with stroke.<br>Second session: Looking at stroke survivors' emotions, thoughts about rehabilitation process and hopes for future.<br><br>Topics:	Data collection :Objective and subjective tests<br><br>Hospital Anxiety and Depression Scale (Zigmond and Snaith,1983) used to evaluate participants' emotional status before and after the intervention.<br><br>A Likert rating scale used after the intervention to elicit feedback from participants regarding the usefulness of the programme for their recovery.<br><br>Participants' contributions were recorded verbatim throughout the group sessions<br><br>Data analysis:<br>Paired sample T-test<br><br>Recordings transcribed, then gathered into themes according to topic and meaning

		Setting: Stroke rehabilitation unit<br><br>Country :UK	Week 1 - "Tell us about your stroke"<br>Week 2 - "How is your rehabilitation going?" and "How do you see the future?"<br>Setting: Stroke Rehabilitation unit (acute phase)<br><br>Provider: Professional-led groups, run by 2 facilitators and a rota of group leaders: 2 speech and language therapists, 1 occupational therapist (OT), 1 physiotherapist (PT) and 1 trainee clinical therapist.<br><br>OT & PT trained by trainee clinical psychologist.<br><br>Length: Total 17 group sessions, each with 3-6 participants, lasting 1-1.5 hours per week for 2-3 weeks<br><br>Cost to participants: Not identified<br><br>Tailoring: Not identified<br><br>Modification: Not identified<br><br>Fidelity assessed: Not identified	
**Hancock (2009)**	Design: qualitative study<br><br>Aim: to examine the experiences of stroke survivors of participating in stroke peer support groups<br><br>Eligibility: all stroke survivors participating in Stroke Recovery/ March of Dimes Canada peer support groups	Sample size: 9 stroke survivors<br><br>Age in years [Range or Mean]: 40-75 Mean=65<br><br>Living conditions: Not reported<br><br>Marital status: Not reported<br><br>Time since stroke: Range < 1-year post stroke- > 9 years	The intervention: Stroke Recovery Canada (local peer support group)<br><br>Developmental rationale:<br>Initiated in response to the need to fill a gap in stroke services and research in Canada, as few supports had been offered to stroke survivors and their families to help them through recovery.	Data collection:<br><br>Semi-structured interviews, lasted an hour<br><br>Aim: Interviews explored the experiences of stroke survivors participating in peer support groups for the following purposes:<br>1) to recognise the experiences of stroke survivors and emphasise the role of peer support in their lives after stroke,<br>2) to increase awareness of peer support groups and their benefits,

		Type/location of stroke: Not reported<br><br>co-morbidity/severity of stroke**,**<br><br>Balance and mobility problems, physical disabilities (usually paralysis of one side of the body), communication and swallowing issues, memory, and mood problems<br><br>Recruitment: sample was recruited from seven local peer support groups; strategy not identified<br><br>Country :Canada	Based on non-medical model, linking stroke survivors with others, aimed at enhancing recovery and developing support network for survivors.<br><br>Content:<br>1) educational topics by speakers, 2) time allocated for group discussion, 3) sharing and 4) recreational activities<br><br>2 groups were developed specially for younger stroke survivors<br><br>Setting: Community<br><br>Provider:<br>6 out of 7 groups led by stroke survivor, care provider (spouse or family member) or a combination of both. Last group coordinated by staff social worker.<br><br>Length:<br><br>Most groups held monthly, but some more frequently for specific programmes, e.g. weekly exercise class.<br><br>Cost to participants: Not identified<br><br>Tailoring: Not identified<br><br>Modification: Not identified<br><br>Fidelity assessed: Not identified	3) to provide opportunities for stories to be shared with other stroke survivors and public,<br>4) to widen stroke research beyond the realm of medical model,<br>5) to enable professionals to gain an insight into experiences of stroke survivors participating in peer support groups in order to inform further initiatives<br><br>Data analysis:<br>Not reported
**Morris & Morris (2012)**	Design:	Participants: Total 18, comprising	The intervention:	Data collection:

	Qualitative instrumental case study<br><br>Aim: to explore the experiences of stroke patients, partners of care and volunteer peer supporters of participating in peer support groups during hospital rehabilitation<br><br>Eligibility criteria<br>All stroke patients hospitalised in the stroke rehabilitation unit and their partners of care. Patients who were bedridden, had severe language or cognitive disabilities or refused to participate were excluded. Participants, including peer supporters, had to participate in at least two sessions to be included in the study.	10 group members (7 stroke survivors(5males& 2 females) & 3 partners of care) and 8 peer supporters (5 former stroke survivors & 3 partners of care)<br><br>Age in years [Range or Mean]: Range 44-95<br><br>Living conditions: Not reported<br><br>Marital status: Not reported<br><br>Time since stroke: Range 3-10 months<br><br>Type/location of stroke:<br>Right sided stroke (n=5)<br>Left sided stroke (n=2)<br><br>co-morbidity/severity of stroke,<br><br>4 had mild and 1 severe dysphasia<br><br>5 had mobility problems (2 used wheelchairs and 3 walking aids)<br>4 had cognitive difficulties (memory)<br><br>Recruitment: Sample strategy not identified<br><br>Setting: Inpatient (stroke rehab unit)<br><br>Country :UK	Hospital-based peer support groups<br><br>Developmental rationale:<br>Based on earlier work on the therapeutic value of peer support groups<br><br>Content:<br>A rolling set of topics agreed by group members (stroke survivors plus their carers) at each session, e.g. moods, recovery. These were discussed in all groups and small groups (divided into stroke survivors and partners of care groups)<br><br>Setting: stroke rehabilitation unit<br><br>Provider: co-facilitated peers (former stroke survivors and partners of care) and 2 staff members. Training sessions completed by peer supporters prior to the group<br><br>Length: total of 13 group sessions twice a week, lasting 1.5 hours<br><br>Cost to participants: Not identified<br><br>Tailoring: speech and language therapists were available when patients required their assistance<br><br>Modification: Not identified<br><br>Fidelity assessed: Not identified	Semi-structured interviews<br><br>Questionnaire<br><br>Seven opened-ended questions and 21 Likert-type items drawn from standardised Therapeutic Factors Inventory (TFI) (Lese & MacNair-Semands, 2000) were administered as semi-structured interviews with stroke survivors and their partner of care and as postal questionnaire for peer supporters<br><br>Aim of the data collection:<br><br>Interviews to increase knowledge of peer support groups by exploring the experiences of stroke patients, carers and peer supporters. The TFI questions were used to gain information related to the group-therapeutic process and examine their links with aspect of participants' experiences that showed in the qualitative analysis of the interviews.<br><br>Data analysis:<br><br>Semi-structured interviews were recorded and transcribed verbatim. Interviews and questionnaire responses were subjected to inductive thematic analysis.

**Kessler et al., (2014)**	Design Qualitative instrumental case study<br><br>Aim<br>To evaluate an individual peer support programme with multiple groups of participants<br><br>Eligibility criteria<br>Admitted to acute care setting after first stroke, showed desire to participate in the peer support programme and had adequate cognitive and communication skills to engage in the interviews (with or without communication aids).	Sample size:<br>Total 38: 16 stroke survivors(75% males), 8 partners of care, 7 peer supporters , 3 program coordinators and 4 HCPs/<br><br>Age in years [Range or Mean]: Mean 64.8 (11.3)<br><br>Living conditions: 4 lived alone, 10 lived with spouse & 2 with other family members<br><br>Marital status: Not reported<br><br>Time since stroke: Acute phase<br><br>Type/location of stroke<br>Recruitment: Type:<br>clot 14 (87.5%)<br>bleed 2 (12.5%)<br><br>Location:<br>left 3 (18.8%)<br>right 13 (81.3%)<br><br>co-morbidity/severity of stroke**,**<br><br>: Barthel Index score<br><br>Stroke survivors<br>(SD), range 78.8 (21.7) = moderate dependency	The intervention: Individual peer support programme<br><br>Developmental rationale:<br>To give newly diagnosed stroke patients the opportunity to meet stroke survivors who had positive experiences of community reintegration.<br><br>Content:<br>Peer hospital visit to newly diagnosed stroke survivors and their partners of care if they were present prior to discharge to introduce hope, followed by post-discharge telephone calls to either stroke survivors or their carers, to provide ongoing support and information on living with stroke and community resources<br><br>Setting:<br>Acute inpatient unit - community<br><br>Provider:<br>Peer-led<br><br>Visits conducted by peers. HCPs provided organizational support. Peers completed in-class group training and shadowed an experienced peer<br><br>Length:<br><br>Total of 17 acute hospital visits and 28 follow-up telephone calls<br><br>1 visit per week, lasting 10 minutes	Data collection:<br>Open-ended semi-structured interview and peer support diaries<br><br>Interviews with stroke survivors and their partners conducted soon after discharge and at 6 months<br><br>Interviews with other participants conducted at one point in time throughout the study.<br><br>The aim of the data collection::<br><br>Interviews to examine the individual peer support programme covered:<br>1) Type of support,<br>2) benefits and harms of peer programme for stroke survivors and their partners of care,<br>4) effect on peer supporters and 5) organisation-related factors.<br><br>Data analysis:<br>Interviews and peer supporters' diaries were recorded, transcribed, and coded following MacPherson and McKie (2010) on the use of qualitative data in programme evaluation

		Setting<br>Acute inpatient unit<br><br>Country: Canada	Telephone calls at 1, 3, 6, 9 and 12 months after discharge, lasting 5-60 minutes<br><br>Cost to participants:<br>No cost identified to participants, but pay was offered to organization staff and HCPs to provide support during the intervention<br><br>Tailoring:<br><br>The 2 peer supporters who carried out the hospital visits were matched to complement each other in terms of strength and severity of disability.<br><br>Modification:<br><br>Initially, post-discharge follow-up calls were made by paid HCPs; however, due to financial constraints, peer supporters made calls to recent stroke survivors whom they visited<br>Fidelity assessed:<br>Not identified	
**Kirkevold et al., (2014)**	**Design** Qualitative feasibility study<br><br>**Aim**<br>To evaluate the intervention focusing on stroke survivors' experiences.<br><br>Multiple case study approach, studying each participant in detail<br><br>**Eligibility**<br>Adults aged 18 and older, had a stroke in the past 8 weeks, medically stable, had adequate cognitive ability to participate and could	Sample size: 25 stroke survivors (17 males & 8 females)<br><br>Age in years [Range or Mean]:<br>Range 33-89<br><br>Living conditions:<br>22 lived at home alone,<br>3 discharged to care home after stroke, one returned home at end of intervention	The intervention :<br>Dialogue-based psychosocial nursing intervention<br><br>Developmental rationale:<br><br>Initiated in response to the need to develop an effective programme to address the psychosocial difficulties after stroke, as earlier research on similar interventions showed modest effect<br><br>Overall aim was to promote psychosocial well-being and adjustment of community stroke survivors	Data collection:<br>Demographic data collected prior to the intervention<br><br>Health professionals' log notes and participant's work sheets collected during the intervention.<br><br>Individual in-depth qualitative interviews (open-ended question with more particular topical questions) held two weeks after the intervention. Interviews with patients having communication difficulties were video recorded<br><br>Aim of the data collection:<br>Log notes and work sheets to describe the process of the intervention.<br><br>Log notes focused on experiences of content, structure and process of each session.

	benefit from the intervention (decided by the physicians and stroke team), wished to participate in the intervention, spoke Norwegian. Aphasic patients were included after their language ability was evaluated by a speech therapist.<br><br>Patients with dementia or severely ill (decided by physicians and stroke team) were excluded.	Marital status: Not reported<br><br>Time since stroke:<br>Approx.4-8 weeks post stroke to 6 months<br><br>Type/location of stroke:<br>Not reported<br><br>co-morbidity/severity of stroke,<br><br>Physical disabilities varied from mild to severe. A majority had moderate effects.<br><br>Many experienced fatigue, vision or hearing impairments, memory and concertation problems. Participants with communication problems suffered from moderate to severe aphasia<br><br>Recruitment: Purposeful sampling<br><br>Setting<br>Hospitals and care homes in three regions of Norway: two large cities and a rural area<br><br>Country: Norway	Based on previous work (systematic reviews of psychosocial interventions, qualitative research and theories addressing psychosocial well-being and coping: Theory of sense of coherence (Antonovsky, 1987), narrative theory (McAdams, 2009; Polkinghorne, 1988) and other philosophies, e.g. guided self-determination (Zoffmann, 2004)<br><br>Content:<br><br>Delivered in 2 formats: individual dialogue with trained HCP (nurse) and group intervention with peers and group facilitators.<br><br>The individual intervention aimed to build therapeutic relationship with each participant, understand each personal situation and identify any issues before they started the group.<br><br>Each session guided by a topical outline covering substantial issues shown in stroke research as particularly relevant to post-stroke trajectories, e.g. relationships, everyday concerns. However, sessions were more open & unstructured, to allow participants to express any concerns.<br><br>Worksheet consisted of prompts relevant to topic of each session, e.g. key words, unfinished sentences<br><br>Setting:<br><br>*Individual intervention*	Work sheets provided information on participants' thoughts, feelings, needs, worries, values, goals and experiences in preparation and throughout the sessions.<br><br>Interviews to explore stroke survivors 'experiences of participating in the intervention<br><br>Data analysis:<br>Interviews were transcribed verbatim. Interviews, log notes and work sheet notes were analysed using qualitative content analysis (Graneheim & Lundman, 2004).

			Pre-discharge: hospital/rehabilitation unit<br>Post-discharge: home or care home<br><br><br>*Group intervention*:<br>Community<br><br>Provider:<br>Professional led.<br>Length:<br>Total 8 sessions over 6 months<br><br>2 initial individual dialogues<br>lasting an hour, followed by 6 group dialogues of 2 hours.<br><br>Two initial meetings held before and immediately after discharge, then every 2 weeks for 2 months and every 4 weeks for the last 2 months<br><br>Cost to participants:<br><br>Not identified<br>Tailoring:<br><br>Assigned to either group or individual intervention based on geographical location of participants and presence of communication difficulties. Participants with aphasia offered only individual intervention.<br><br>Trained staff with good knowledge of assisting those with communication difficulties facilitated sessions for participants with aphasia<br><br>Planned topics were changed if a participant desired to discuss a topic that differed from that assigned for a specific session.	

			Modification:<br><br>The intervention delivered to aphasic patients had to be prolonged, as topics planned for each session could not be covered.<br><br>Fidelity assessed:<br><br>Facilitators completed 16 hours of training prior to the intervention and were supervised throughout the intervention in individual and group supervision meetings.<br><br>Trained facilitators who delivered intervention to aphasic participants were under supervision throughout the intervention by speech therapists	
**Muller et al.,(2014)**	Design<br>Pre-post longitudinal study in three stages: programme development, implementation and evaluation.<br><br>Aim<br>To explain development, results and assessment of the YESS group for younger stroke survivors<br><br>Eligibility<br>Stroke patients 18-65 years old, cognitively intact and able to sustain attention during a 60-90-minute session, able to physically attend the sessions and to communicate	Sample size:<br>13 stroke survivors all under the age of 65 years old, ranged from 24-60<br>10 males and 3 females<br><br>Living conditions:<br>Not reported<br><br>Marital status:<br>6 single, 5 married, 1 had partner, 1 divorced<br><br>Time since stroke:<br><br>Range: < 1 year - 8 years	The intervention:<br><br>Young empowerment stroke support (YESS) programme, no control group<br><br>Developmental rationale:<br>Aim: to offer support and education to stroke survivors aged 65 and under.<br><br>Three theoretical perspectives guided establishment and implementation of the YESS programme:<br>1-Group dynamic theory (Yalom & Leszcz, 2005) provided the overall format, using the "therapeutic mechanism of change" within the group context.	Data collection:<br><br>Objective test<br><br>Survey<br><br>The aim of the data collection:<br><br>1) Stroke impact scale (SIS) (Duncan et al., 2001) to detect changes after stroke. including emotional, social factors and coping<br><br>2) Community integration questionnaire (CIQ) (Dijkers, 2000) to detect level of participation and activity after stroke within home environment and community.

	verbally or with a communication aid.<br>Participants who required physical assistance were allowed to participate only if family members were present.	(n=5) > 1<br>(n=4) 1-2<br>(n=3) 3-5<br>(n=1) 6- 8<br><br>Type/location of stroke:<br>Not reported<br><br>co-morbidity/severity of stroke,<br><br>Not reported<br><br>Recruitment<br>Sample strategy not identified<br><br>Country: U.S.A	2-Social learning theory (Cole & Tufano, 2008) guided the application of the intervention, using the value of peer interaction, active participation and cognitive behavioural methods within groups.<br>3- Model of human occupation (Kielhofner,2008) was used to actively experiment, model and repeat new behaviours within groups. This approach, with in-group feedback, is effective in promoting new performance and roles.<br><br>Content:<br>Needs assessment survey of younger stroke survivors determined the key group models, e.g. change in mood, decrease in socialization<br><br>Setting:<br><br>Rehabilitation-based hospital programme (outpatient)<br><br>Provider:<br><br>Professionally led by occupational therapist and other HCPs. Only one session facilitated by peer (stroke survivor)<br><br>Length:<br><br>13 peer support groups<br><br>Nine twice-monthly, 90-minute sessions over 18 weeks<br><br>Cost to participants:<br>Participants had to arrange their own transport.	Participants completed both outcomes prior to the first the group and again at 9th session (end of the intervention)<br><br>3) Member satisfaction survey to evaluate the outcomes of the intervention and to elicit more specific information on how group process and modules meet the intervention's objectives that SIS and CIQ did not detect. Administered at the end of intervention<br><br>Data analysis :<br><br>Wilcoxon ranked sum test.

			Participants who needed physical assistance could attend only if a family member was present.<br><br>Tailoring:<br>The duration of each session was chosen to lessen potential cognitive and physical limitations<br><br>Modification:<br>Not identified<br><br>Fidelity assessed:<br>Not identified	
**Corsten et al., (2014)**	**Design** : case series (pre and post rest design , with follow up evaluation at three months with no intervention)<br><br>Research type :<br>Expletory<br><br>**Aim**:<br>To develop and assess of an "interdisciplinary multi-model of biographic -narrative approach to enhance QoL in people with post stroke aphasia<br><br>**Eligibility :**<br><br>A phasic stroke patient with no sever disorder in speech perception	**Sample size:**<br>17 participants with chronic post stroke aphasia (9 males and 8 females)<br><br>**Age in years [Range or Mean]:**<br><br>Mean: 63.59 years<br><br>**Living conditions:**<br>Not reported<br><br>**Marital status**: Not reported.<br><br>co-morbidity/severity of stroke**,**	**The intervention:** adopted biographic-narrative intervention for stroke survivors with post chronic aphasia<br><br>**Developmental rationale:**<br><br>The biographic-narrative intervention based on the following theories<br><br>(1) Socio-cultural theories (Bauer et al., 2008; MacAdams, 2008).<br>(2) Social -construction -oriented theories<br>(3) Based on the idea that stroke can impact on the Quality of Life (QoL) as it alters sense of self and ability to return to previous activities, routine and roles which all contributed to an individual's identity. Re-constructing sense of	**Data collection:**<br><br>Quantitative measures ((self-administered)were completed during the week before the intervention (baseline), the week post intervention and at 3 months follow up . The assessment of self-reported states of mood using VAMS were administered at the end of first interviews and the last interviews and at the end of first and last group session<br><br>1) Health Related Quality of Life (HRQL) measured by Aachen life Quality Inventory(ALQI) (Engell et al., 2003)<br>2) Satisfaction with life measured by Satisfaction with Life Scale (SWLS) (Diener et al. 1985).<br>3) Mood measured by the German version of Visual Analog Mood Scale (VAMS) (Stern,1997)

	and depression symptoms according to The Aachen Aphasia Test (Huber et al., 1983) and the Geriatric Depression Scale(Sheik and Yesavage, 1986).	All participants had chronic aphasia, but varied type. Severity of aphasia ranged from mild to moderate.<br><br>**Time since stroke:**<br>Average :40.82 months post stroke<br><br>**Type/location of stroke**:<br>Apart from 2 participants, all had aphasia as result of left C.V.A<br><br>**Recruitment**:consecutive sampling<br><br>**Settin**g: ambulant rehabilitation unit and aphasia support groups<br><br>Country: Germany	identity and re-created sense of meaningfulness in life are essential to QoL (Clarke and Black, 2005)<br>(4) Narrative therapy utilizes stories as a methods of re-constructing sense of self and facilitate coping process.<br>(5) The aim of the intervention: to enhance QoL, reinforce identity-re-negation, social participation and sense of self-competence<br><br>**Setting:**<br><br>At ambulant rehabilitation units and at the Catholic University of Applied Sciences in Mainz<br><br>**Content**:<br>Total: 12; 5 face to face interviews and 7 group sessions over 10 weeks.<br><br>The first three face to face interviews were designed as a narrative interview (unstructured in-depth interview) aimed at attaining identify re-negation. The first part of the interview aimed at providing opportunity to tell personal stories without interruption. The second part of the interview included question related to any issues were raised during the initial narration<br><br>The last 2 interviews were in the format of semi-structured interviews aimed at exploring issues and to prepare for group sessions in particular with people with sever aphasia.	Qualitative data was conducted by semi-structures interviews with questions aimed at focusing on personal growth and change in identity .The interview was conducted at baseline to discover participants' expectation while at post intervention and at 3 months follow up , the interviews aimed at detecting if life perception and sense of self-esteem had changed after participation in in the intervention.<br><br>**Data analysis :**<br><br>For all quantitative measured ,Wilcoxon ranked sum test was and paired *t*-tests were used<br><br>The qualitative data was analyzed based on ground theory (Bryant & Charmaz,2007)

			The group sessions were designed to promote learning from peers and enhancing social interaction .The groups' theme presented were related to health or leisure activities .For each group subject, managing the past, present, and future was the focus.<br><br>**The length:**<br>Total 12 (5 face to face interviews and 7 group sessions (each group included 5-7 participants), lasted maximum of 90 minutes over 10 weeks. One interview was conducted per week over the first three weeks. The group sessions started at the second week and over three weeks, one session was conducted per week. During week 4, only one group session conducted. There was beak (no intervention) at week 5 and 8. At week 6 there were one interview and one group sessions. The following week (week 7) there was only one group session. During week 9, there were 1 group sessions and one interview and the last week (week 10), there was only one group session.<br><br>The face to face interviews were begun first to provide opportunity to tell life story. There were breaks at week 5 and 8 to enable participants to integrated narrative experiences into their daily life perspective. Interviews 4 and 5 were aimed at preparing members for group sessions	

			**Provider:**<br>Professional led by 2 facilitators<br>speech therapist and pedagogue. Both had experiences in dealing with language disorders as well as knowledge of biographic approach. The facilitators who ran the group session introduced the group themes and promoted group discussion.<br><br>**Modification:** the intervention utilized biography work that especially modified for people with aphasia<br><br>**Tailoring**<br>Intervention (narrations) was supported by multi-mode; strategies such as pictures writing, pictograms in case of sever aphasia<br><br>one to one support help from speech therapist was provided to participants with severe case of speech production<br><br>Cost to participants:<br><br>Not identified<br><br>Fidelity assessed:<br>Not identified	

**Corsten et al., (2015)**	**Design** : Mixed methods design with pre and post test<br><br>**Research type** : Expletory study<br><br>**Aim**:<br>To provide a quantitative and qualitative evaluation of the change in QoL in-stroke survivors with post stroke chronic aphasia after participating in an adopted interdisciplinary biographic-narrative intervention<br><br>**Eligibility** :<br>Stroke survivors with no sever speech disorder and depression symptoms according to The Aachen Aphasia Test (Huber et al., 1983) and the Geriatric Depressiob Scale Short Form (Sheik and Yessvage, 1986).	**Sample size:**<br>27 post stroke aphasia patients<br>15 males and 12 females<br><br>Age in years [Range or Mean]:<br>Mean, 60.85 years<br><br>co-morbidity/severity of stroke**,**<br><br>Severity of aphasia ranged from mild to moderate<br><br>Broca's, 5 Anomia, 3 Wernicke's, 2 global and 9 others<br>Depression were not assed as it was an exclusion criterion<br><br>**Type/location of stroke:**<br>All participants expect from 3 had aphasia as result of left cerebrovascular accident<br><br>**Time since stroke**:<br>Mean: 60.82 months<br><br>**Living conditions:**<br>Not reported<br><br>Marital status: Not reported	**The intervention**: structured biographic-narrative intervention for stroke survivors with post chronic aphasia<br><br>**Developmental rationale:**<br><br>The biographic-narrative intervention based on the following theories<br><br>(6) Socio-cultural theories (Bauer et al., 2008; MacAdams, 2008).<br>(7) Social -construction -oriented theories<br>(8) Based on the idea that stroke can impact on the Quality of Life (QoL) as it alters sense of self and ability to return to previous activities, routine and roles which all contributed to an individual's identity. Re-constructing sense of identity and re-created sense of meaningfulness in life are essential to QoL (Clarke and Black, 2005)<br>(9) Narrative therapy utilizes stories as a methods of re-constructing sense of self-identity ,making sense of the illness experience ,re-constructing sense of purpose and meaningfulness in life and identify won strengths and available resources which could assist people in managing personal and environmental difficulties (Brody, 1994) .	**Data collection:**<br><br>Quantitative measures (self -assessment) , participants were promoted by the researcher by reading out each question and written down answered<br><br>Assessments were completed before week 1(baseline), the week post intervention and at 3 months follow up . The assessment of self-reported states of mood using VAMS were administered at the end of first interview and the last interviews and at the end of first and last group session.<br><br>4) Health Related Quality of Life (HRQL) measured by Aachen life Quality Inventory(ALQI) (Engell et al., 2003)<br>5) Short term affective states of mood measured by the German version of Visual Analog Mood Scale (VAMS) (Stern,1997)<br>6) Cognitively based long-term Life satisfaction measured by with Satisfaction with Life Scale (SWLS) (Diener et al. 1985).<br><br>**Aim of the data Quantitative measures:**<br>To discover change in QoL over the time<br><br>**Qualitative data :**<br><br>Semi-structured interviews conducted at the week post intervention , lasted 10-15 minutes sung a topic guide

		Setting: ambulant rehabilitation unit and aphasia support groups<br><br>Recruitment<br>: consecutive sampling<br><br>Country: Germany	**Content:**<br><br>5 face to face in-depth interviews and 7 group sessions (each group included 5-7 participants).<br><br>To provide an opportunity to tell own stories , the face to face interview was initiated first and the group sessions started in the second week, both were conducted weekly with one week break at week 5 and 8 in order to give participants time to incorporate narrative experiences into their everyday life perspective. One interviews and 2 groups sessions conducted following each break.<br><br>The first three face to face interviews were designed as a narrative interview (un-structured in-depth interview) aimed at attaining identify re-negation. The participants were given opportunities to tell their life stories without interruption.<br><br>The last 2 interviews were in the format of semi-structured interviews aimed at exploring issues.<br><br>The group sessions were designed to promote learning from peers. enhancing social interaction and support identify shaping. The groups' theme presented were related to health or leisure activities to facilitates self-reflection on identity, exploring one's self and any strategies that could help in managing the new or changed situation. For each group subject managing the past, present, and future was the main focus. At the beginning of each	Aim of the data collection :<br><br>Semi-structured interviews aimed at exploring the mechanism of the intervention that underpin identify development and its effect on QoL. The main questions focused on the participant's experiences<br>Of participating in the intervention and their emotions after the intervention. Additionally, there were questions related to change in identify and the affect on everyday life<br><br>**Data analysis**:<br><br><u>For quantitative data</u><br><br>Wilcoxon ranked sum test<br><br>paired *t*-tests were used<br><br><u>Qualitative data</u><br><br>The interviews were taped, video recorded, transcribed orthographically according to the principles of Du Bois (1991) and coded. The qualitative analysis was guided by interpretative principle from ground theory (Corbin and Strauss ,2008). 2 researchers analyzed the qualitative data .

			group session, each member contributed to the topic<br><br>**The length:**<br>One session per weeks<br>Five face to face interviews and 7 group session over 10 weeks, lasted 90 minutes<br><br>**Provider:**<br>Professional led by speech therapist and a education professional with expertise in biographic approach. The facilitators who ran the group session introduced the group themes and promoted group discussion<br><br>S**etting**: at university<br><br>**Cost to participants:**<br><br>Not identified<br><br>M**odification:**<br>the intervention utilized biography work that especially modified for people with aphasia. The size of the group had to be modified due to 2 members missed the first session and 3 missed the last session<br><br>**Tailoring:**<br>The face to face interviews was carried out by one of the facilitators, whereas the group	

			sessions were conducted by both. However, in case of sever aphasia, the interviews were carried out by the speech and language therapists. Additionally, during both format of the intervention, assisting with communication skills was provided as needs<br><br>Augmentative communication and one to one support help form speech therapist were provided to participants with severe case of speech production were provided with<br><br>**Fidelity assessed:**<br>Not identified	
**Clark et al., (2018)**	Design: Qualitative<br><br>Aim: To gain insight into stroke survivors' thoughts on group self-management intervention and how it could be delivered<br><br>Eligibility<br>Stroke survivors with one or more strokes, able to be interviewed verbally (patients with communication difficulties were included if they understood a two-step command and were able to express thoughts) and receiving care in a UK stroke care pathway	Sample size<br>14 stroke survivors (6 males and 8 females)<br><br>Age in years [Range or Mean]:<br><br>Mean 58, range: 47-78<br><br>Living condition:<br><br>Alone (n=6),<br>with spouse (n=6),<br>with spouse and daughters (n=2)<br><br>Marital status :<br><br>Not identified<br><br>Type/location of stroke:	The intervention: Specific group self-management programme for stroke survivors prior to attendance<br><br>Developmental rationale:<br><br>Based on previous work on the importance of addressing long-term unmet needs of stroke survivors by providing opportunity to access SMPs, in particular group-based. A feature of group delivery is to offer a place where similar experiences can be shared, reciprocal gain, reinforcement of self-management skills and many other values.<br><br>Content:<br>No details provided<br>The length:<br>Not identified	Data collection:<br><br>Semi-structured interviews<br><br>Aim of the data collection:<br>1) To explore survivors' experiences of self-management during their individual stroke journey in order to determine when/where to offer the group in the recovery journey/stroke path<br><br>2) To explore values, ideas and difficulties of the groups in order to gain insight into possible benefit and challenges<br><br>Data analysis :<br><br>In line with qualitative research guidelines (Braun, 2013), data collection and analysis were conducted simultaneously.<br><br>Data management used NVivo 10 (SPSS Inc, Chicago, IL)

		Not reported<br><br>Time since stroke:<br><br>Range: 4 - 174 months post stroke<br><br>co-morbidity/severity of stroke,<br><br>Permanent impairments (mood problems, motor dysfunctions and weakness, poor balance, fatigue and aphasia; 1 had mild expressive aphasia<br>Recruitment<br>Consecutive sampling from the Sobell Stroke Database at Queen Square<br>obell stroke database at Queen Square<br><br>Setting<br>National Hospital for Neurology and Neurosurgery, Queen Square, London<br><br>Country: UK	The provider:<br>Not identified<br><br>The setting:<br>Not identified<br><br>Modification :<br>Not identified<br>Tailoring:<br>Not identified<br><br>Fidelity assessed:<br>Not identified	
**Chow (2018)**	Design Double blind RCT<br><br>Aim<br>To evaluate the effectiveness of group narrative therapy among Chinese stroke survivors and compare them with treatment as usual (psycho-educational approach)<br>Eligibility	Sample size :192 stroke survivors<br><br>Males : Intervention group 61.46%<br>Control group 62.50%<br><br>Age in years<br>[Range or Mean]<br><br>Means: Intervention group 72.49, control group 72.84<br><br>Living conditions	**The intervention:**<br>Narrative therapy (NT) group intervention, with control group<br><br>Development rational<br>Based on earlier work on the effectiveness of NT in improving well-being in different populations.<br><br>The theoretical basis was social constructionism and narrative therapy	**Data collection:**<br>Objective measures<br><br>Seven outcome measures taken at baseline, 1, 2- and 4-months post intervention:<br><br>1) 10-item self-esteem scale (Rosenberg, 1965)<br><br>2) Pearlin Mastery Scale to measure to what extent participants felt their life outcomes to be under their control (Pearlin & Schooler, 1978).

	Adults aged 60 years and older, diagnosed with stroke over the past 1-2 years, had completed hospital or rehabilitation-based programme, mobile with or without mobility aids, not experiencing current psychological distress, mental health or psychotic problems, intact hearing, able to carry out minimal daily activities, scoring 18 or more on Cantonese Chinese Mini-Mental State Examination, intellectually intact, not diagnosed with personality disorder, no history of suicide or violent behaviour and agreed to meet the expectations of the group	Alone:<br>intervention group 14%, control 11%<br><br>With others:<br>intervention group 81%, control 85%<br><br>Marital status:<br><br>Married: intervention group 67%, control 73%<br><br>Significant others:<br>intervention group 29%, control 23%<br><br>Time since stroke:<br>1-2 years post stroke<br><br>Type/location of stroke:<br><br>Ischemic stroke intervention group (83.72%)<br><br>Control group (81.82%)<br><br>co-morbidity/severity of stroke,<br><br>Initial MMSE mean scores:<br>intervention group 26.11;<br>control group 26.24<br><br>Only 8% of participants in both groups had some extent of speech problems	Content:<br>Narrative therapy within group. It included therapeutic conservation between therapist and participants by asking therapeutic questions, participants shared stories<br><br>Setting:<br>Community<br><br>Provider:<br><br>Facilitated by narrative therapist<br><br>Length:<br><br>*Intervention group*<br>2 hours per week of NT group sessions for 8 sequential weeks<br><br>*Control group*<br>Treatment as usual: psycho-educational sessions on same schedule.<br>Cost to participants:<br>Not identified<br><br>Tailoring:<br>Not identified<br>Modification:<br>Not identified<br><br>Fidelity assessed:<br><br>Trained staff who delivered the control intervention group were supervised by HCP to ensure fidelity.<br><br>Fidelity of intervention group was not discussed.	3) Herth Hope Index to measure survivors' hopefulness, overall acceptance of stroke as a chronic illness and post-stroke life (Herth, 1992).<br><br>5) Chinse Geriatric Depression Scale Short Form to measure depression (Lee et al., 1993).<br>6) Life Satisfaction Scale-Chinese to measure satisfaction with various needs of older stroke survivors (e.g. relationships, health) (Lou et al., 2008).<br><br>7) Stroke knowledge; eight self-reported items to assess survivors' subjective understanding of their conditions (e.g. symptoms, rehabilitation) (Chow, 2008)<br><br>Data analysis:<br>SPSS 23 Windows

		Recruitment<br>Cluster, selected randomly from stroke registries of five regionals clusters of the Hong Kong Hospital Authority<br><br>Country: China (Hong Kong)		
**Masterson-Algar et al., (2018)**	Design<br>Mixed methodology on co-design principles (Batalden et al., 2016; Clarke et al., 2017).<br><br>Aim<br>To construct and evaluate a novel peer-led coaching intervention to improve meaningful leisure and social participation following stroke.<br><br>**Eligibility criteria**<br>All stroke patients who had been hospitalised with stroke within the previous 6 months and had been discharged home. Patients discharged to care homes were excluded.	Sample size :<br>Phase 1: 79 stroke survivors,<br>Phase 2: 18 stroke survivors and 10 family members,<br>Phase 3: 5 stroke survivors (2 males and 3 females)and 5 peer supporters(<br><br>Age in years<br>[Range or Mean]<br><br>Range :<br>38-60<br><br>**Living conditions**<br><br>2 with spouse who was key source of support;<br>2 alone<br><br>1 with teenage children & limited support from family<br><br>Marital status:<br>Not reported<br><br>Time since stroke:	The intervention:<br><br>Peer-led coaching intervention (individual-based), with no control intervention<br><br>Development rational:<br><br>Initiated in response to the need for a peer-led intervention to improve meaningful social and leisure participation, as professional-led intervention had little effect.<br><br>Based on peer coaching model, utilising "transformational leadership and performance profiling", based on personal construct theory (Kelly, 1955)<br><br>The peer coaching model is based on: 1) exchange between peers and stroke survivors could offer motivation for participation; 2) peers offer "experiential knowledge"; 3) sharing stories allows peers to relate to stroke survivors, make connections and offer empathy.<br><br>Content:	Data collection:<br><br>1) Quantitative measures<br><br>2) Interviews<br><br>The aim of the data collection :<br><br>Quantitative measures to evaluate function (Barthel Index), mood (General Health Questionnaire v12), and participating in leisure (Individualised leisure profile), social activities (Stroke impact scale) at 6 months post stroke.<br><br>Interviews with stroke survivors and family members on their experiences related to leisure and social participation at 6 months post stroke.<br><br>Interviews with stroke survivors and peer supporters to evaluate the intervention from their perspectives<br><br>Data analysis:

		Within 6 months post stroke<br><br>Type/location of stroke:<br>Right parietal infraction (n=1)<br>Lacunar infraction (n=1)<br><br>Left frontal infarct (n=1)<br><br>co-morbidity/severity of stroke,<br><br>Not reported<br><br>Recruitment<br>Sample strategy not identified<br><br>Setting<br>Stroke survivors and family members were recruited from stroke registry of three sites. Peer supporters were recruited from local stroke groups and local newspapers<br><br>Country: UK	Face-to-face sessions with a trained peer to identify and attain social and leisure-related goals.<br><br>“Peer handbooks”<br>were provided to support training and the conduct of the intervention.<br><br>“Stroke survivors’ handbook” gave information related to the intervention, outline of its goals and content of each coaching session.<br><br>Setting:<br>Community: public places, e.g. coffee shop, library<br><br>Provider:<br><br>Peer-led by former stroke survivors who completed training workshops (4 days in 3-hour class sessions, over 2 weeks.<br><br>Length:<br><br>Maximum of 6 one-hour sessions<br><br>Cost to participants:<br>Not identified<br><br>Tailoring:<br><br>Peers were matched with stroke survivors based on post-stroke impairment and geographical location.<br><br>Setting of the coaching sessions determined by participant’s preference	Numerical data analysed on SPSS v22.<br><br>Interviews digitally recorded and transcribed, following constructivist grounded theory (Charmaz, 2002)

			Modification:<br>Not identified<br><br>Fidelity assessed<br>Peers were in regular contact with the researcher during the intervention for ongoing support.	
**Christensen et al., (2019)**	Design: Qualitative study<br><br>Eligibility criteria<br>Stroke survivors and partner of care who attended peer support groups	Sample size :32 in total: stroke survivors and their partners of care (9 males and 23 females)<br><br>Age in years<br>[Range or Mean]<br>Average 67 years<br><br>Living conditions<br>Not reported<br><br>Marital status:<br>78% married, 13% divorced, 6% widowed, 3% single<br><br>Time since stroke:<br>In the community<br><br>Type/location of stroke:<br>Not reported<br><br>co-morbidity/severity of stroke,<br><br>Not reported<br><br>**Recruitment:**<br>Sample strategy not identified	The intervention:<br>Stroke peer support group<br><br>Development rational:<br><br>Stroke survivors and their partners of care received and provided social, emotional, affirmational and information support through peer support groups. The sharing of experiences enhanced understanding of others<br><br>Content:<br><br>The sessions consisted of presentations of educational topics with time allocated for discussion.<br><br>Setting:<br>Community<br><br>Provider:<br><br>One group facilitated by a formal stroke survivor, one by a nurse who was also the main carer of a stroke survivor and the	Data collection :<br>Focus group sessions using a semi-structured discussion guide and open-ended questions, lasting 30-60 minutes<br><br>Sessions held over two months (from October to December 2016) at the same time and locations as the scheduled groups.<br><br>The aim of the data collection :<br><br>Focus groups using open-ended questions to allow participants to engage in discussions in order to explore the psychological, informational and social benefits of participating in stroke peer support groups to stroke survivors and their partner of care<br><br>Data analysis :<br><br>Focus group were audio recorded, transcribed verbatim and analysed using an inductive coding approach and thematic content analysis (Hsieh & Shannon, 2005).

		**Setting**<br>Four rural North Carolina stroke support groups<br>Country : U.S.A	remaining groups by nurses who had experience of patient education.<br><br>Length:<br><br>4 sessions of 1- 2 hours once a month or every 2 months, for 6 to 12 years. A majority had not attended since the programme began.<br><br>Cost to participants:<br>No cost to participants identified<br>Tailoring:<br><br>Not identified<br>Modification:<br>Not identified<br><br>Fidelity assessed:<br>Not identified	
**Appalasamy et al., (2020a)**	**Design** : mixed methods research<br><br>A parallel group randomised controlled trail (RCT), with control group (who received standard care (patient education, counselling with neurologist , outpatient appointment, self-monitoring check, information on stroke prevention and medication)<br><br>Single blind randomised controlled trail	**Sample size**: 54 stroke patients (more than 50% males than females in both groups)<br><br>**Age in years:**<br>Mean :56 years for control group and 53 for the intervention group<br><br>**Living conditions:**<br>Not identified<br><br>**Marital status:**<br>Not identified	**The intervention**: face to face video narrative presentation + hospital standard care<br><br>**Development rational:**<br><br>The video narrative intervention based on the social learning theory, health belief model construct and the motivational promotes to encourage stroke survivors to develop self-efficacy skills<br><br>**Content:**	**Data collection :**<br><br>Baselines sociodemographic data , medical information collected at baseline (T0)<br><br>**Quantitative measures** :quantitative measures were collected at baseline(T0) and after the study during 3 months following up period (T1)<br><br>Main outcome measures are the following<br>(1) Assessing understanding and taking medications as well as knowledge and perception by MUSE at baseline (T0) and for 3 months follow up (T1) to detect change through the intervention and comparing the results with the control group

	The intervention group received (standard care + video narrative intervention .<br><br>Semi-structured interview<br><br>**Aim:**<br>To assess the feasibility and acceptability of study process . Additionally, to gain preliminary results of the effect of video narrative on medication understanding and use self-efficacy(MUSE) and blood pressure control<br><br>**Eligibility**:<br><br>All adults aged 18 and over , diagnosed with their first stroke within 6 months of the recruitment phase, attended the Neurology Outpatient Department of Hospital in Kuala Lumpur(HKL), were taking stroke risk preventative medications prescribed by (HKL) and could give consent and comprehended the English and Malay language<br><br>Post stroke survivors with depression symptoms and cognitive impairments were excluded	**Time since stroke:**<br>Not identified<br><br>**Type/location of stroke:**<br>Majority of the participants (80%) had ischemic stroke<br><br>**]** co-morbidity/severity of stroke**,**<br><br>Most of the participants had many stroke risks factors such as hypertension.<br>50% had diabetes, almost 90% were prescribed al least 3 types of stroke preventative medications<br><br>**Recruitment:**<br><br>selected randomly from outpatient neurology clinic at HKL<br><br>**Country:** Malaysia	A real model stories (a story of a neurologist and a stroke survivors) in order to show real emotions , while focusing on the importance to adhere to stroke preventative medication<br><br>Stories were culturally appropriate<br><br>Subtitles and short quotes were integrated to enhance attentiveness and comprehension of the purpose of the videos<br><br>**Provider:**<br>A researcher met each participant individually for conducting assessment or video viewing<br><br>**Setting:** at a neurology outpatient clinic<br><br>**Length:**<br>The intervention period over 3 months<br><br>**Cost to participants:**<br>The one of the most common causes of dropout from study was fear of increasing stress<br>The outcome measure (questionnaire) administered were time-consuming and exhaustive<br><br>**Tailoring:**<br>Not identified<br><br>**Modification:**<br><br>Culturally appropriate messages<br>Provided in both English and Malay langue to mee the need of participants	(2) To evaluate participant's knowledge of stroke and associated treatment measured by the Stroke Knowledge Test (SKT)<br>(3) To assess patient's perception of their illness and belief towards their medications measured by Brief Illness Perception Questionnaire (BIPQ) and Brief about Medicine Questionnaire (BMQ).<br>(4) To assessed perceived psychical and mental health by Short Form (36) Health Survey<br><br>The feasibility of the study processed was evaluated by determining the recruitment , retention, and completion rate of the study was assessed at baseline (T0)<br><br>In addition, participant's feedback on the burden of the trial process and outcomes measures<br><br>**Qualitative data :** semi-structured with consent participants from the intervention group (phone interview, lasted 10 minutes)after completion of the trail (2 weeks post (T1) to obtain participants' feedback and opinions regarding the acceptability of the intervention<br><br>**Data analysis :**<br><br>Quantitative Data<br><br>IBM SPSS<br><br>Qualitative Data<br><br>8 Phone Interviews<br><br>12 Written feedbacks

			Questionaries were translated form English to Malay languages. pilot trail was connected with 5 participants and experts before the actual trial to ensure content validity.<br><br>**Fidelity assessed:**<br>Validation of the intervention procedures and the content of the video narrative intervention had been assessed in previous study (Appalasamy JR et al., 2019)	The interviews were recorded and transcribed verbatim and coded<br><br>The interviews transcript and written feedback of participants were analysed suing thematic analysis<br><br>Software NVivo 11 was used to assist in identifying theme and organising coder
**Appalasamy et al.,**<br><br>**(2020b)**	**Design :**<br><br>A single blind Randomised Controlled Trail (RCT) for 12 over 12 months , with control group (hospital standard care)<br><br>**Aim :**<br>To investigate the effectiveness of video narratives integrated with Health Belief construct on Medication Understanding and use Self -Efficacy (MUSE(and its associated aspects in stroke survivors<br><br>**Eligibility :**<br>Adult aged 18 and over, diagnosed with their first stroke within 6 months of the recruitment phase. Post stroke patients who prescribed antihypertension and antithrombotic medication, had satisfactory level of literacy, and could comprehend English and Malay languages. Post stroke	**Sample size:** 216 stroke patients **(**There were almost 20% more men participants than women in both groups)<br><br>**Age in years**<br>**[Range or Mean]**<br>Ranged from 20 and 90 years old<br><br>**Living conditions**<br><br>Not identified<br>**Marital status:**<br>Not identified<br><br>**Time since stroke:**<br>Not identified<br><br>**Type/location of stroke**<br><br>More than 90% of participants had ischemic stroke.<br><br>co-morbidity/severity of stroke**,**	**The intervention**: 2 sets of video narratives (in English and Malay language) presented face to face + hospital standard care (such as speech therapy, rehabilitation, medication, and nutritional consultation ,appointment with neurologists and outpatient appointment ,self-monitoring check and information on stroke treatment and medication)<br><br>**Content:**<br>Hospital standard care +motivational video narratives of a stroke patient experiences in successfully managing<br>the illness and a neurologist perspective on the illness and treatment integrated with behavioral construct of health believe mode to promote stroke survivors' self-efficacy related to medication understanding.<br><br>The video included motivational and short culturally appropriate messages and quotes that emphasized on self-efficacy in particular self-managing medications and increased awareness of stroke risk factors.<br><br>**Development rational:**	**Data collection** :<br><br>Sociodemographic data was collected at baseline<br><br>Outcomes were administered at baseline(T0), at 6 months (T2) and at 12 months (T4)<br><br>**Quantitative measures:**<br><br>(1) The primary outcomes to detect change in participant's self-efficacy perception in understanding and taking medications measure by MUSE .<br>(2) To evaluate participant's knowledge of stroke and associated treatment measured by the Stroke Knowledge Test (SKT)<br>(3) To assess patient's perception of their illness and belief towards their medications measured by Brief Illness Perception Questionnaire (BIPQ) and Brief about Medicine Questionnaire (BMQ).

	patients with depression symptoms and cognitive impairment were excluded.	hypertension, 60% had diabetes and several had may stroke risk factors and more than 80%<br>were prescribed al least 3 types of stroke preventative medications<br><br>**Recruitment:**<br><br>selected randomly from outpatient neurology clinic at HKL<br><br>**County:** Malaysia	Based on the idea that self-efficacy is the essential factor that influence positive health behaviors and proactivity. Self-efficacy has been integrated in several educational interventions, however, the interventions had mixed findings due to heterogeneity of the population and methodology. Additionally, due to the lack of emotional and cultural elements.<br><br>On the other hand, narrative stories might offer an effective mechanism in influencing viewers as they actively absorbed with the stories or information presented.<br><br>Moreover, behavioral modification studies showed that people engage and focus more effectively via visual and hearing stimulus<br><br>Based on these evidences, the researcher team aimed at investigating the effectiveness of motivational video narratives, integrated with behavioral construct of the health belief mode on self-efficacy of medication understanding and use among post stroke patients.<br>.<br><br>**Setting:** at Hospital Kuala Lumpur, Malaysia (HKL)	**Data analysis:**<br><br>IBM SPSS

			**Provider:**<br><br>A stroke patient and a neurologist<br><br>**Length:**<br><br>The intervention was offered at baseline (T0), at 3 months (T1) and at 6 months (T2)<br><br>**Cost to participants:**<br>The one of the most common causes of declining to participate in the study was fear of increasing stress<br><br>**Tailoring:**<br><br>Not identified<br>**Modification:**<br><br>Culturally appropriate messages<br>Provided in both English and Malay langue to mee the need of participants<br><br>The transcripts were initially written in English and then translated to Malay language. A linguistic expert validated the content of the translated transcript. Additionally, doctors, educational experts, pharmacists and stroke survivors reviewed and edited the transcript and videos several times<br><br>**Fidelity assessed:**	

			Validation of the intervention procedures and the content of the video narrative intervention had been assessed in previous study.	

Table S3 The Completed 13- item modified COREQ framework (Soundy et al .,(2016) adapted from the 32-item COREQ framework (Tong et al., 2007)

**Domain 1: Research team and reflexivity**			**Hancock (2009)**	**Morris & Morris (2012)**	**Kirkevold et al., (2014)**	**Kessler et al., (2014)**	**Clark et al., (2018)**	**Christensen et al., (2019)**
Personal Characteristics		*Scoring*	*ID for CerQual: Study 10*	*ID for CerQual: Study 2*	*ID for CerQual: Study 9*	*ID for CerQual: Study 1*	*ID for CerQual: Study 3*	*ID for CerQual: Study = 7*
1	Interviewer/facilitator Which author/s conducted the interview or focus group?	If they have identified the individual give a point, if they haven't give 0, if you unclear type U.	0<br>U<br>Unclear who did the interview	0<br>U<br>Unclear who did the inte5rview	1<br>*"Members of the research team, who had not delivered the intervention and whom the participants did not know, interviewed the participants without language problems, allowing them to more openly voice criticism and concerns regarding the intervention. For participants with aphasia, the same person conducted both the intervention and the interviews"* Page 5 of 12	0<br>Unclear who did the interview	1<br>clear who did the interviews	1<br>clear who did the interviews

2	Experience and training. What experience or training did the researcher have?	Where experience is clearly detailed or a detailed reference to training OR experience is made a point is given. IF unclear or absent give zero.	0<br>no details of experience or training given	0<br>no details of experience or training given	1<br>*With regard to the research team, all researchers conducting this study had a nursing background, were women and were trained as qualitative researchers within nursing science. . They had different clinical experiences. Three of the researchers had conducted previous qualitative studies of experiences following a stroke, one specifically focusing on persons with aphasia"*<br>Page 6 of 12	0<br>no details of experience or training given	1<br>details of experience or training given	0<br>no details of experience or training given
Relationship with participants								
3	Relationship established, Was a relationship established prior to study commencement?	Give a point if details of how the researcher or person who undertook qualitative data collection met individuals, identified any previous relationship. Where this is unclear type U. Where this information is absent type 0.	0<br>Not mentioned	0<br>Not mentioned	*1*<br>*Local recruiters in the hospital or home care service approached potential participants; the recruiters judged whether the patients met the inclusion criteria" Page 5 of 12*<br>*"Participants provided written, informed consent to a person outside the research group before being included **"page 6 of 12***	0<br>U<br>Unclear	1 details of how the researcher or person who undertook qualitative data collection met individuals were given	0<br>Not mentioned

			Members of the research team, who had not delivered the intervention and whom the participants did not know, interviewed the participants without language problems, allowing them to more openly voice criticism and concerns regarding the intervention". "criticism and concerns regarding the intervention. For participants with aphasia, the same person conducted both the intervention and the interviews Their substantial communication difficulties required continuity in the relationship and familiarity with the intervention process to elicit the participants' experiences and thought" . page 5- of 12			

4	Participant knowledge of the interviewer. What did the participants know about the researcher? e.g. personal goals, reasons for doing the research	A point is scored where information about what the participants knew about the research they were being invited to participate in was mentioned. This includes sending background information and study information sheets. Score zero where this information is absent and U where this information is unclear	*1* *"Participants were provided information about the research project through their peer support groups (in meetings or through newsletters) and all participants volunteered their time"(page No.4)*	0 Not mentioned	*1* *"Local recruiters in the hospital or home care service approached potential participants; the recruiters judged whether the patients met the inclusion criteria, provided written and oral information about the study and collected informed consent" page 5 of 12*	*1* *" all the peer supporters and coordinators who were involved in the peer support program and health who worked on the hospital unite where program Offred were informed about the project by the researcher during a scheduled meeting and invited to contact the researcher if they were interested in participating "* *Page 3 of 9* *"all new stroke survivors and their care partner (when present) who received visit as part of peer support program between February and October 2012*	*0 Not mentioned*	*1* *"The group leaders told regular participants when a focus group would be happening as part of their meeting and those who were interested attended. Participation was voluntary, and survivors and caregivers were not required to attend together. Written consent was obtained from all participants after explaining the purpose of the study" page 3*

						were invited to participate in the study by a peer support during the visit if the professional judged that they met the inclusion criteria"<br>*Page 2 of 9*		
5	Interviewer characteristics. What characteristics were reported about the interviewer/facilitator? e.g. Bias, assumptions, reasons and interests in the research topic	Score a point where information about the characteristics of the interviewer, their bias, interests or reasons for participating in research are identified. Score zero where this information is absent. Score u where this information is unclear.	0<br>Not mentioned	0<br>Not mentioned	*1*<br><br>*"All authors are nurses with a clinical interest in stroke rehabilitation"*<br><br>*Page 11 of 12*<br>*"The authors declare that they have no competing interests"*<br>*Page 11 of 12*	1<br><br>*" this study was carried out at the request of the agency providing this service. While member of the research team do not have any direct affiliation with SSAO, 2 have worked with the agency on previous evaluation projects and therefore do have relationships with the agency and some of its*	1<br>information about the characteristics of the interviewer, their bias, **interests** or reasons for participating in research are identified<br><br>*"All the semi-structured interviews [35] were conducted by EC who is a PhD student working*	0<br>Not mentioned

				employees. While the research team made a conscious effort to conduct the analyses and present the finding an objective manner, the find may be biased towards more positive outcomes as result of this relationships" page 8 of 9	*within the context of stroke self-management" page 570*<br><br>*"EC is the Trial Manager of this study" Page 575*	
**Total Score for Domain 1.**	**1/5**	**0/5**	**5/5**	**2/5**	**4/5**	**2/5**
**Domain 2: study design**						
Theoretical framework						

6	Methodological orientation and theory. What methodological orientation was stated to underpin the study? e.g. grounded theory, discourse analysis, ethnography, phenomenology	Score a point where the paradigm and methodology are given. Score zero where both aspects are missing. Score U where this information is unclear.	0<br>Mot mentioned	*1*<br>*"Seven open-ended questions and an additional 21 Likert-type items taken from the Therapeutic Factors Inventory (TFI [29]) were presented by semi-structured interview (recipients), or questionnaire (peer supporters). The transcripts of recipients and peer supporters were analysed separately,* ***using inductive thematic analysis*** *[30], to identify superordinate and subthemes encompassing aspects of the participants' experience. The Likert items were analysed descriptively to establish "consensus" by identifying items that most participants rated in the same way. This was performed across all participants since numbers were small. The relationship between the content of these items and the themes emerging from the thematic analysis was then examined qualitatively to determine if the items could be assimilated into the*	1<br>paradigm and methodology are given	1<br>Methodology was given	1<br>the paradigm and methodology are given	1<br>methodology are given.

				themes. Any such items were reported with the relevant theme" (page no 348)				
7	Non-participation. How many people refused to participate or dropped out? Reasons?	Score a point where the number and the reason or attempts to identify the reason are given (e.g., a point is score if they say participants would not give a reason for non-participation). Score zero where this information is	0<br>numbers are not identified with reasons	0<br><br>Clear number without reasons	1<br><br>Clear numbers are identified with reasons	1<br>Clear numbers are identified with reasons	1<br>number and the reason or attempts to identify the reason are given	0<br>numbers are not identified with reasons

		absent and score u where this information is unclear.						
**Data collection**								
8	Interview guide. Were questions, prompts, guides provided by the authors? Was it pilot tested?	Score a point where testing of the interview script is identified either as a pilot or as a way to determine the content and accuracy of items used. **Score a point where consideration to the derivation of questions have come from**.	*1*<br>*reference to development of the interviews was made.*<br>*"The framework of the study and the interview questions were informed by stroke research as well as literature concerning social and peer support"(page no,3)*	*1*<br>*"semi-structured interviews with seven open-ended questions: e.g. "Is there anything at all you would have liked to have been different about the groups?"; "How has your life changed by participating in the groups, if at all?"; "Is there anything you learned in the groups that you are now using?". Recipients were encouraged to expand on their answers, and this format provided focus whilst allowing individuals to provide detailed, individualised information [31]. There were also 21 Likert-type items taken from the TFI [29]*<br>*Page 349*<br>*Seven open-ended questions and an additional 21 Likert-type items taken from the Therapeutic Factors Inventory (TFI [29* *Page348*	1<br>testing of the interview script is identified	1<br>consideration to the derivation of questions have come from.	1 piloting of the interview script<br>consideration to the derivation of questions have come from	1<br>consideration to the derivation of questions have come from

9	Field notes - Were field notes or reflective diary made during and/or after the interview or focus group?	Score a point where field notes are identified. Score zero where field notes are not identified. Score U where this information is unclear.	0.No mention of field notes.	0.No mention of field notes.	*0*<br><br>*No reflective diary of field note identified during or after the interviews*	*1*<br><br>*"six interview transcripts and dairies were read by the primary investigator and research assistant".*<br><br>*"data collection took place over 10 months. A total of 27 acute care visits diaries (5 peer supporter, 2 volunteer program coordinators) were collected. As well, post discharge telephone contact diary record were obtained for 28 stroke survivors*<br>*Page 4 of 9*	0.No mention of field notes	0.No mention of field notes.
10	Data saturation. Was data saturation discussed?	Score a point where saturation of data is considered. Score a point where another form of sample size reference is	0.No mention of sample size	0.No mention of sample size	0.No mention of sample size	1<br>Saturation is considered	1<br>data saturation mentioned	0.No mention of sample size

		made. Score U where this information is unclear.						
**Total for Domain 2**			**1/5**	**2/5**	**3/5**	**5/5**	**4/5**	**2/5**
**Domain 3: analysis and findings**								
Data analysis								
11	Description of the coding tree. Did authors provide a description of the coding tree?	Score a point if an audit trail is given. **Score a point if a coding tree is mentioned** or score a point if another technique is mentioned that provides a way to structure the information gained.	0 No detail given	1<br>Audit trail is given *"Several of the transcripts were read by a second member of the research team. This enabled the themes to be discussed, verified and modified and provided an assessment of the validity of the themes [31]. Two themes were merged and another re-labelled as a result of this process"*<br>Page 349 &Page 350	1<br>Coding tree mentioned	1<br>*"Document of each stage of analysis were kept to create an adult trail."*<br>*"analytic grid that used to organise information critical to the research objective".*<br>Page 4 of 9<br>coding scheme used	1<br>coding tree is mentioned	0 No detail given

				Coding tree mentioned				
12	Derivation of themes Were themes identified in advance or derived from the data?	Score a point if data driven or theory driven **coding is identified or if it is clear how analysis was determined**.	0<br><br>Framework not mentioned	1<br><br>Framework mentioned	1<br>Theme driven from data	*1*<br><br>*MacPherson and McKie's recommendation for the use of quantitative data in program evaluation were followed* "page 4 of 9	1<br>Framework mentioned	1.Framework mentioned.
Reporting								

13	Clarity of minor themes Is there a description of diverse cases or discussion of minor themes?	Score a point if there is sub-detail for each major theme. Score no points where this information is absent or u if this information is unclear.	0 Subthemes not given	1 Subthemes given	1.Subthemes given	1 There is sub-details of each major theme	1 Subthemes given in details	0 Subtheme did not given
**Total for domain 3**			**0/3**	**3/3**	**3/3**	**3/3**	**3/3**	**1/3**
Grand total			**2/13**	**5/13**	**11/13**	**10/13**	**11/13**	**5/13**

Table S3a Grade CerQual Ratings of Concern for methodological limiations

Concern criteria	**Hancock (2009)**<br>**10**	**Morris & Morris (2012)**<br>**2**	**Kirkevold et al., (2014)**<br>**9**	**Kessler et al., (2014)**<br>**1**	**Clark et al., (2018)**<br>**3**	**Christensen et al., (2019)**<br>**7**
1 Methodological Limitations<br>The extent to which there are concerns about the design or conduct of the primary studies that contributed evidence to an individual review finding:	Serious	Moderate	No or minor	No or minor	No or minor	Moderate

Table S4: Summary assessment of the overall risk of bias for quantitative studies (Higgins & Green, 2011)

**Trial*<br><br>and type**	**Components of risk of bias/key risk criteria**						**Summary within trial**	**Comments on high risk components**	**Classes of evidence(Burns et al., 2012**
	1	2	3	4	5	6			
**Chow (2011)**<br><br>RCT	L	H	L	H	L	H	H=3<br>L=3<br>U=0	**Selection bias**<br>No identification of allocation concealment<br><br>**Attrition bias**<br>Number who dropped out stated, but no reasons provided and no discussion of how this would impact on analysis and results.<br><br>**Other bias**<br>Sample size not justified and no protocol.	***Level of evidence***:<br>**II**<br><br>***Risk of bias***:<br>moderately low<br><br>***Design classification***:<br>moderate quality RCT<br><br>*Criteria*:<br>Moderate because of (a) violation of allocation concealment; (b) co-interventions applied equally through randomisation; (c) follow-up rate
**Masterson-Algar et al., (2018)**<br><br>Mixed methodology	H	H	H	L	L	L	H=3<br>L=3<br>U=O	**Selection bias**<br>No identification of random sequence generation or allocation concealment.<br><br>**Detection/performance bias**<br>No blinding of assessors indicated<br><br>**Other bias**<br>No protocol; however, sample size was considered	***Level of evidence***:<br>**III**<br><br>***Risk of bias***<br>Moderately high<br><br>***Design classification****:*<br>Moderate cohort study<br><br>*Criteria*<br>Moderate, because of (a) violation of random sequence generation or allocation concealment; (b) violation of blind or independent assessment.

**Appalasamy et al., (2020a)**<br><br>Mixed methods research	L	L	H	L	L	L	H=1<br>L5	**Detection/Performance bias:**<br>blind **or** independent assessment for important outcome was not ensured	***Level of Evidence:***<br>*II*<br><br>***Criteria :***<br>*Moderately low risk*<br><br>***Design classification:***<br>*Moderate quality RCT*<br>*because the following:*<br>*(a)violation of Blind or independent assessment for important outcomes*
**Appalasamy et al., (2020b)**<br>Single blind RCT	L	L	U	L	L	L	L=5<br>U=1	**Detection/Performance bias:**<br>Unclear if blind **or** independent assessment for important outcome was ensured	***Level of Evidence:***<br>*II*<br><br>***Criteria :***<br>*Moderately low risk*<br><br>***Design classification:***<br>*Moderate quality RCT*<br>*because the following:*<br>*(a)violation of Blind or independent assessment for important outcomes*

**Gurr (2009)**<br><br>Mixed methodology, including qualitative themes	H	H	H	H	L	H	H=5<br>L=1<br>U=0	**Selection bias**<br>No identification of random sequence generation or allocation concealment.<br><br>**Detection/performance bias**<br>No blinding of assessors indicated<br><br>**Attrition bias**<br>No attempt to follow up on participants and no data on participants who dropped out in the analysis. No intention-to-treat analysis mentioned<br><br>**Other bias**<br>No protocol; no sample size identified; unsatisfactory response rate; questionable appropriateness of outcome measure	***Level of evidence*:**<br>**IV**<br><br>***Risk of bias*:**<br>High<br><br>***Design classification***<br>Case series<br><br>*Criteria*<br>High risk because of (a) violation of random sequence generation or allocation concealment; (b) violation of blind or independent assessment; (c) failure to follow up and absence of intention-to-treat analysis
**Muller et al., (2014)**<br><br>Pre and post study, with no control group	H	H	H	H	L	H	L=1<br>H=5<br>U=0	**Selection bias**<br>No identification of random sequence generation or allocation concealment.<br><br>**Attrition bias**<br>No follow-up of participants who discontinued group<br><br>**Detection/performance bias**<br>No blinding of assessors indicated<br><br>**Other bias**<br>No protocol mentioned and sample size not considered	***Level of evidence***:<br>**IV**<br><br>***Risk of bias*:** High<br><br>***Design classification*:**<br>Case series<br><br>*Criteria*<br>High risk because of (a) violation of random sequence generation or allocation concealment; (b) violation of blind or independent assessment; (c) failure to follow up

**Corsten et al., (2014)**<br>Mixed methods	H	H	H	H	L	H	H=5<br>L=1<br>U=0	**Selection bias:** No identification for sequence generation , or that is was randomised. no allocation concealment identified<br><br>**Detection/Performance bias:** all quantitative assessments were carried out by a researcher who did not carried out the intervention , however, was not blind to purposes of the assessments. The assessments were self-administered to minimise the influence of the assessor<br><br>**Attrition bias**: they gave the number of people who dropped out, but reasons were not provided and there was no discussion how this would impact on analysis and result<br><br>**Other bias: :** No protocol mentioned, and sample size not considered	***Level of Evidence:***<br>***IV***<br><br>***Risk of bias:***<br>*High risk*<br><br>***Design classification :***<br>*case series*<br><br>***Criteria :***<br>*High risk because the following:*<br>*(a)violation of random sequence generation or allocation concealment;(b) violation of blind or independent assessment;(c) failed to follow up and no intention to treat analysis conducted*

**Corsten et al., (2015)**<br>Mixed methods	H	H	H	H	L	H	H=5<br>L=1<br>U=0	**Selection bias**: No identification for sequence generation no identification, or that is was randomised. Or no allocation concealment identified<br><br>**Detection/Performance bias:** all quantitative assessments were carried out by a researcher who did not carried out the intervention , however, was not blind to purposes of the assessments . The Qualitative data was collected by speech therapists who were " neural interviewers(no blinding of assessors mentioned)<br><br>**Attrition bias** they gave a number of people who dropped out, but reasons were not provided and there was no discussion how this would impact on analysis and result<br><br>**Other bias:** No protocol mentioned, and sample size not considered	***Level of Evidence:***<br>*IV*<br><br>***Risk of bias:***<br>*High risk*<br><br>***Design classification:***<br>*case series*<br><br>***Criteria :***<br>*High risk because the following: (a)violation of random sequence generation or allocation concealment;(b) violation of blind or independent assessment;(c) failed to follow up and no intention to treat analysis* ***conducted***

Trial* donated by first author.Risk of bias criteria: 1, Selection bias=random sequence generation; 2, selection bias=allocation concealment; 3, Detection/performance bias= blinding of personnel, assessors and participants; 4, Attrition bias=incomplete outcome data; 5, Reporting bias=short-term selective outcome reporting; 6, Other bias=potential threats to validity, e.g. consideration of protocol.

Level of risk of bias: H, high risk of bias ;U, unclear risk of bias ; L,Low risk of bias .

Table S5 synthesis stage 1 presenting examples of the qualitative tubulisation

Study	Result	Comment by R.A
**Kessler et al., (2014)**	Type of support provided (source of data from all categories)<br><br>Data collected from the diaries and interviews with the new stroke survivors and peer supporters identified that emotional support was provided during the initial in- hospital visit in the form of hope, encouragement, and re- assurance. Emotional and affirmational support occurred through taking time to listen, sharing of stories and validation of feelings.<br><br>I honestly didn't want them to leave because I just wanted to continue talking to someone who actually had gone through what I went through. (New Stroke Survivor-Sylvie, initial interview)<br><br>Informational support was also offered during the initial and telephone visits. Peer supporters provided written as well as verbal information on resources in the community including those offered by SSAO. During the initial visit this information tended to be more general in nature. However, during follow-up telephone calls, information was targeted to the new stroke survivor's or care partner's needs and included	1-Emotional support during the **initial on-site visit by the peer supporters** (outcomes for stroke survivors) in from of hope, encouragement and reassurance<br>2- Affirmation support (Outcomes) **during the initial onsite visit by peer supporter**<br><br>Mechanism of psychosocial benefit; (a) taking time to listen and sharing stories, (b) validation of feeling.<br><br>- Informational support (Outcome s for stroke survivor's s) **during the initial on-site visit and follow up telephone calls**.<br>Form and type of information provided by peer supporter.<br>Form: provided written as well as verbal<br>. Types a) Initially during the on-site visits the type of information was general, b)during the follow up was targeted to meet the needs of new stroke survivors and their carers (outcomes for stroke survivor's and care

	information on specific services avail- able in the community. Information to assist with both finding and accessing services was provided to six out of the 28 people for whom follow-up diaries were kept.<br><br>Benefits of peer support for the stroke survivors and their care partners (source of data from all categories)<br><br>All groups identified benefits for the new stroke survivors and their care partners. The emotional support provided was seen as beneficial at a time when stroke survivors and care partners were feeling overwhelmed by the unknown. The visit from the peer supporters encouraged and motivated the stroke survivors to work towards recovery. After having someone who had gone through a similar experience take time, listen, share experiences, and make a connection, stroke survivors reported feeling validated and less alone. Information received from a peer regarding the experience of living with stroke was generally given more value than that received from a healthcare professional.<br><br>And they [peer supporters] provided sort of reassurance... and provided a real face. When you're dealing with doctors and nurses they're great.... But they're medical people and they can talk to you about what you've just gone through, but the chances are more than likely that they haven't... The people in Stroke Survivors, well they have. (New Stroke	partner) information included finding and accessing specific service available in the community.<br><br>visit from the peer supporters encouraged and motivated to work towards recovery (psychological positive outcome for new stroke survivors) **Mechanism of psychosocial benefit** 1- relatedness, 2- taking time tom listen 3- sharing experiences 4 connection , 4- feeling validating 5- belonging (‘ less alone)<br><br>Informational support from peer about living with stroke was more value than that provide by health professionals.<br><br>**Mechanism of this** might be Mechanism might be relatedness and reassurance.

	Survivor-Mike, initial interview)<br><br>Peer supporters were also seen as a source of inspiration by some stroke survivors who expressed an interest in pursuing a peer supporter role in the future.<br><br>Care partners also benefitted from the emotional support. They reported feelings of reassurance and decreased isolation particularly from the follow-up phone calls.<br><br>It reassures me, you know, they ask "How is [name of partner]? Can we do anything for you?" This is very important. (Care Partner-Liz, 6 month interview)<br><br>A few care partners reported that they had read the information kit and found it helpful.<br><br>But it's nice to have all those resources [in the SSAO information kit] that you can contact because you're overwhelmed sort of, the person is really ill and you're wondering what they are going to need. (Care Partner-Sue, initial interview)<br><br>Harms of peer support for the stroke survivors and their care partners (source of data form new stroke pt,care partner, health professional, program coordinator_)<br><br>No specific harms of the visits were identified by the new stroke survivors. However, potential harms were	Considered as source of inspiration by some stroke survivors (positive outcome for new stroke survivors) and created interest to taking new role in the future.<br><br>emotional support in particular **during the follow up calls** (positive outcome for care partner)as the feel reassurance and decrease isolation.<br><br>**Mechanism** for care partner by offering help.<br><br>Few care partners found information kit helpful .<br><br>**Mechanism** : convenience/ available resource to contact when you feel overwhelmed and uncertain about the need of ill relative.<br><br>No negative outcome of the visit of peer supporters on newly diagnosed patient No specific harms of the visits were identified by the new stroke survivors.

	identified by one care partner, one health professional and one coordinator. The care partner felt that her partner was not ready to receive this type of visit because his condition had not stabilized enough for the message of hope to be perceived as realistic.<br><br>Yeah I thought it was maybe a little bit premature. .. He wasn't sleeping well, he wasn't medically stable... he wasn't really ready for someone to tell him everything was going to be all right because it wasn't all right. (Care Partner-Joanne, initial interview)<br><br>It is interesting to note that the new stroke survivor in this situation did not voice such concerns.<br><br>The health professional raised concern about potential harms when patients with mild stroke were visited by peer supporters with more obvious physical disabilities. This health professional and one of the coordinators re- ported receiving feedback from a few new stroke survivors that being visited by someone with a significant visible disability (such as someone using a wheelchair) was up- setting. The Program Coordinator reported that she had received calls to SSAO expressing this concern.<br><br>Two calls. .. from people that we visited on the acute care floor [informed us] that they did not appreciate a person in a wheelchair coming in for the visit,. ..And to have somebody come in in a wheelchair could make	One car partner, one health professional and one coordinator identified potential harm of the visit of the peer supporters **WHY** one care partner felt<br>**a)** the patient was NOT ready (too early) to receive this type of visit (the initial one site visit) and message of hope due unstable condition of the new stroke pt.<br>**b)** Unrealistic<br><br>However, new stroke survivor in this situation did not voice such concerns.<br><br>One health professional and one coordinator raise a concern by a few stroke patients with mild or less disability when visited by peer supports with more severe obvious disabilities (e.g. wheelchairs users). Negative outcome for stroke patients (2 calls for complain)= Upsetting regarding on site visit<br><br>Why because the give them a negative message and an anticipation about the future.

	them feel as if they're not going to be able to walk again. (Coordinator-Carol)<br><br>New stroke survivors and healthcare professionals also expressed concern about the number of peer visitors present during a visit. When there is a new peer sup- porter being trained the number of peer supporters may increase from two to three. Feedback indicated that one- on-one interactions were preferable, having two peer supporters visit was acceptable, but any more could be overwhelming for the stroke survivor.<br><br>Impact of peer role on the peer supporter (peer/ program coordinator)<br><br>All peer supporters and coordinators indicated that offering support to fellow stroke survivors was beneficial to the peer supporter. While concern was expressed by program coordinators about the potential emotional im- pact on peer supporters for whom the visit may trigger past feelings related to the initial experience of stroke, none of the peer supporters interviewed described experiencing these types of feelings. A few peer supporters reported concern about their ability to do a good job and be understood, particularly with new stroke survivors who had more severe impairments or significant aphasia.<br><br>I’m not always comfortable going into a room maybe if somebody has really severe disability, and if they	New stroke patients and health professionals reported that too many peer supporters on one visit at the acute site was not preferable – individual one to one is preferable.<br><br>2 peer supporters were acceptable on one visit and more than 2 was overwhelming.<br><br>positive impact of the role of peer on **All** peer supporters and coordinators and they reported that offering support was beneficial to them.<br><br>Program coordinators (not being expressed by stroke peer supporters themselves) potential emotional harm on peers **WHY** trigger experience relate to their initial stage of condition.<br><br>**A few** peer supporters raised concern about the ability to do good job as peer and being understood.<br><br>**Why** less comfortable and did not known what to do especially when new stroke patient is severely

	can’t talk or they’re really upset or... I don’t know what else to say. (Peer Supporter-Charles)<br><br>Some people you go and see, they’re so deeply depressed, and some of them that has aphasia that can’t speak to you, it makes me feel real sad. (Peer Supporter-Steve)<br><br>Another concern expressed by one peer supporter was the feeling that since he had no visible disability, he may lack credibility among new stroke survivors who did not believe that he had experienced a stroke.<br><br>Another, more frequent concern expressed by peer supporters was their ability to remember procedures and routines. Due to the fact that peer supporters may only be scheduled once per month, some peer supporters re- ported feeling “bothered” about their ability to remember required details.<br><br>For me like six weeks is like forever and I just forget the whole, I forget all my training. .. Not all my training but important parts. (Peer Supporter-Charles)<br><br>Peer supporters who used the local adapted transportation service had the additional frustration of sometimes arriving late and missing the visits.<br><br>Personal benefits noted by peer supporters included increased social connections, personal growth, enjoyment and the feeling that they had been able to	disabled, unable to communicate and deeply depressed ,the peer supporters felt sad.<br><br>**one peer supporter** expressed concern (negative) felt lack of credibility **Why** not having visible disability and new stroke patients might think he or she did have stroke.<br><br>**more frequent** concern expressed by peer supporters their ability to remember, and feeing bothered to remember details procedure and routine .**Why** only scheduled once a month to dot the onsite visits.<br><br>frustration by peer supports who used the adapted transportation **Why** as they sometimes arrived late and missing the visits.<br><br>Several personal benefits of the weekly on site vists reported by peer supporters including; **1)** increase social connection and interaction, 2**)** increase personal

	make a difference in the lives of others. Following the weekly visits, the<br><br>Page 6 of 9<br><br>peer supporters would go for coffee which provided an opportunity for social interaction, support, and peer mentoring. Several peer supporters reported that while the visits posed challenges mentioned above, they were able to push themselves, and build coping skills and confidence, all of which contributed to their personal growth. Positive feedback received from new stroke survivors reinforced a sense of purpose - a sense that they had contributed.<br><br>Perceived processes required to offer such a program (challenges all categories)<br><br>The processes involved in setting up the peer support program involved close collaboration with the healthcare team to negotiate the referral process, the type of information to be provided to stroke survivors by the peer supporters, and duration of visits. Processes were put in place to ensure that privacy and confidentiality of patient information were protected, and that safety concerns for peer visitors were addressed. An important facilitator for the peer supporters was that parking costs were covered by the hospital.	growth **3)** feel of enjoyment **4)** feeling of usefulness 5) peer mentoring<br><br>**Several** peer supporters while the visits posed challenges, however, **1)** they were able to motivate themselves **2)** build coping skills **3)** build confidence **4)** increase personal growth **5**) positive feedback form the new stroke patients led to reinforce sense of purpose **6)** sense of contribution.<br><br>**<u>The process of setting up</u>** the peer programs required the following :**1)** close collaboration with the health care professional at the acute setting to negotiate the referral process **2)** type of information to be provided by the peer supporters **3)** duration of the onsite visit **4)** process to ensure the privacy and confidentiality of the patient's information **5)** process to ensure the safety of the per supporters.<br><br>**Facilitator** of the peer program ;**1)** peer supporters parking cost covered by hospital, **2)** commitment of health care professional despite of their busy load to complete the referral, **3**) health professional also

	Commitment on the part of healthcare professionals who were willing to take the time to complete the referrals despite busy caseloads was a **key** program component. These professionals also provided important on- going collaboration to address program challenges and improve service to stroke survivors. While data was being collected both the referring professionals and the program coordinators expressed an interest in developing feedback mechanisms following initial visits. Two important goals for these mechanisms were to ensure that all eligible patients were seen, and to ensure health professionals were aware of any issues brought up during the visits that they could help address.<br><br>The recruitment, training and orientation of the peer supporters were also identified as critical to program success. Training provided coordinators with a method of ensuring all peer supporters were aware of the pro- gram mandate and procedures. The peer supporters also noted that the training provided them with an opportunity to develop communication skills and start building confidence in their new roles.<br><br>At first it was new ... I didn't know if I was going to be able to do it [volunteer role], to feel like I could contribute and then I learned that yes I could, that it was good, and that gave me the courage I needed to keep on [volunteering]. (Peer Supporter-Michelle)	provide ongoing collaboration to address program challenges and improve service to new stroke.<br><br>referral health professionals and coordinators initiated the follow up calls to **Why** 1) to ensure all the eligible new patients were seen **,2)** to know the feedback of on site visit and if there were issues occurred during the visit they could help to address.<br><br>**4)** training and orientation of the peer support was a critical to program success. WHY help peer to be aware of program mandate and procedures and provide the peer supporters with chance to develop communication skills and building confidence in new role.

	Common preferred characteristics of peer supporters were identified by all categories of participants. These were being authentic, friendly, confident, a good listener, knowledgeable regarding resources and programs and respectful of the stroke survivor. As noted earlier, visibility of the peer supporter's disability had a negative<br><br>impact on some stroke survivors but promoted the feel- ng of shared experience for others.<br><br>The support offered via telephone calls post discharge from hospital was felt by program coordinators and peers supporters to be an important resource for stroke survivors. However, these follow-up calls posed many challenges. Primary among these was the ability to reach people once they left hospital. Although the coordinators reported reaching 82% of the stroke survivors whom they visited in hospital, they felt that the people they could not contact may have been those most in need of support. Additionally, while it was originally hoped that the peer supporter who visited the stroke survivor in hospital would complete the follow-up call, many of the peer supporters did not feel that they had the knowledge and skills to identify and provide the needed support once stroke survivors left the hospital. As well, the organizational skills required to track calls until each in- dividual was reached were a challenge for peer sup- porters with cognitive deficits. Peer supporters noted that organizing call-backs took a lot of effort and that it was difficult to stay on top of information regarding available services. Coordinators noted the need to re- mind peer supporters to make calls. For this reason, the majority of telephone follow-up visits were completed by the Program Coordinator	Common preferred characteristics of peer supporters **1)** friendly,**2)** authentic,**3)** knowledgeable of resources and program ,**4)** good listener 5) confident<br>**Hinder: 1)** having no visible disability<br><br>**6)** peer supporters and coordinators felt that the support offered to new stroke patients by the follow up calls were useful source.<br><br>**Hinder:2)** peer supporters and coordinators felt that it was challenging to do the follow up calls because people could not reach after being discharged from hospital, although 85% was reached during the follow up<br><br>it was hoped that all the peer supporters who did the initial visit on site would do the follow up calls however, they was not the cause because **many** felt that they ,**4)** did not have the knowledge and skills to identify and provide support to new patients when the left hospital ,**5)** they did not have the organisation skills to track calls until the each patient reached especially with peer supporters with cognitive disability and it was time consuming and required a lot of effort **,6)** difficulty to be on the top regarding available resources in the community and 7) coordinator noted they need to remind peer to make calls, therefore they majority of the phone calls conducted by coordinator, however when finical stopped, peer need to made the calls

Table S6 Synthesis stage 2 an example of the descriptive analysis undertaken.

<table>
<tr><th>Study</th><th>Result</th><th>Comment by R.A</th></tr>
<tr>
<td>Chow (2018)</td>
<td>
Preliminary analyses
The demographic information and clinical characteristics of sample participants at baseline shown in Table 1 reveals that, of the N=192 original participants, 33 (17.19%) dropped out
throughout the data collection time. More than half the participants in the intervention group (61.46%) and the TAU group (62.50%) were male. The mean age of the stroke survivors in the intervention and TAU groups were M=72.49 (SD: 7.27) and M=72.84 (SD: 7.82) respectively. The majority of participants suffered an ischaemic stroke (intervention: 83.72%, TAU: 81.82%). Initial MMSE scores were similar in the two groups (intervention: 26.11%; TAU: 26.24%). No other significant differences between the intervention and the TAU group were found for all clinical characteristics and selected demographic information. All selected outcome variables at baseline showed no significant differences between intervention and TAU groups (Table 2); thus both groups were deemed comparable at baseline.
Analyses of intervention and control groups
Repeated measures ANOVAs were conducted to compare the patterns of outcome change over time between the two groups. These results are presented in Table 3. In the intervention group, significant time effects were demonstrated across all 7 outcome measures. However, for the control group, a significant time effect was only found in the meaning in life (F (3, 246)=3.28, p=0.02), hope (F (3, 237)=3.15, p=0.03), self-esteem (F (3, 243)= 2.70, p=0.49) and stroke knowledge (F (3, 234) = 43.35, p<0.001). <u>The noted change patterns over time were further investigated in their mean plots, which suggested that the patterns of change over time in the outcome measures across the intervention and the TAU groups were statistically different</u>. <u>Further, many of these positive effects were sustained at 4 months post intervention, in the intervention. Pairwise comparisons revealed that hope, meaning in life, self-esteem and stroke knowledge had significant changes between T0 and T1, T0 and T2, and T0 and T3</u> (Table 3).

<u>To further delineate intervention effectiveness, regression of change between (1) T1 and T0, (2) T2 and T0, (3) T3 and T0 were conducted. These analyses are displayed in Table 4 and indicate there is a delineated pattern: NT shows a higher improvement in most outcome scores over the conventional psycho-education intervention. Results further indicate that the intervention group significantly improved in mastery (R2=0.04, F (1, 174) = 7.12, p = .05), hope (R2=0.04, F (1, 176)=7.24, p = .05), meaning of life (R2=0.04, F (1, 172)=7.04, p=0.01) and life satisfaction (R2=0.04, F (1, 178)=6.72, p= .05), at post intervention versus baseline</u>. <u>Improvement of self-esteem was marginally significant </u>between interim and baseline in the intervention group R2=0.02, F (1, 179)=3.60, p=0.06<u>. Regarding stroke knowledge, a negative mean difference though insignificant seemed to be understandable, suggesting the relative effects of NT and the psycho-education group are similar.</u> We posit that the reason is the delivery of psycho-education mainly provides information about stroke during the rehabilitation process.
Finally, the results of these analyses support the contention that NT is beneficial in reconnecting the persons’ sense of mastery, enhancing self- esteem, and reconstructing meaning of life with hope in one’s life, (i.e., ascribing more positive meaning to a critical life event) while having other positive influences on their depression and satisfaction of life

Table 2: Means, Standard Deviations and Baseline Differences between Groups of Outcome Variables Over Time (N=192).
<table>
<tr><th>Variable Tested</th><th>T_0</th><th>T_1</th><th>T_2</th><th>T_3</th><th>Baseline Difference Between Groups</th></tr>
<tr><td>Intervention Group</td><td>M (SD)</td><td>M (SD)</td><td>M (SD)</td><td>M (SD)</td><td>t-test</td></tr>
<tr><td>Hope</td><td>6.13 (1.23)</td><td>6.62 (1.35)</td><td>6.89 (1.28)</td><td>6.75(1.58)</td><td>t(189) = -1.14, p = .25</td></tr>
<tr><td>Meaning in Life</td><td>3.47 (.65)</td><td>3.77 (.62)</td><td>3.93 (.59)</td><td>4.33 (.71)</td><td>t(189) = -1.73, p = .86</td></tr>
<tr><td>Mastery</td><td>3.39 (.85)</td><td>3.60 (.77)</td><td>3.88 (.86)</td><td>3.85 (.77)</td><td>t(188) = -1.18, p = .24</td></tr>
<tr><td>Self-Esteem</td><td>2.60 (.44)</td><td>2.85 (.44)</td><td>2.99 (.32)</td><td>2.94 (.48)</td><td>t(189) = -1.57, p = .12</td></tr>
<tr><td>Depression</td><td>.32 (.25)</td><td>.33 (.27)</td><td>-.25</td><td>-.20(.26)</td><td>t(190) = 1.44, p = .15</td></tr>
</table>
</td>
<td>
Stroke survivors in the intervention showed significant improvements in many psychological outcomes including meaning of life, mastery , self -esteem , hope, and life satisfaction after participating in the narrative group sessions. Furthermore, most of the positive outcomes in the intervention group sustained up to 4 months post intervention. Stroke survivors in the group did not show similar improvement across most of the outcome measures.

Improvement of self-esteem was only significant between interim and baseline in the intervention group R2=0.02, F (1, 179)=3.60, p=0.06

Negative mean differences were noted in the stroke knowledge outcome although insignificant between the intervention group and the control group. This could be explained by the control group received psychoeducation intervention which focused on providing informational support related to stroke and recovery form stroke
NT group: (T0), M(SD)=3.26(2.15), (T1)=5.05(1.88), (T2)=5.49(2.09), (T3)=5.54(2.17); ontrol group: (T0)= 3.46(2.41), (T1)=5.45(1.90), (T2)=5.76(1.93), (T3)=5.86(2.17)

insignificant increase in depression was found form baseline to one month (estimated effect size 0.05). However, a significant reduction at 2 months (estimated effect size 2.24) and four months (estimated effect size 2.04).
</td>
</tr>
</table>

			(.26)		
Life Satisfaction	.71 (.19)	.78 (.17)	.88 (.15)	.84 (23)	*t*(190) = -.62, *p* = .54
Stroke Knowledge	3.26 (2.15)	5.05 (1.88)	5.49 (2.09)	5.54 (2.17)	*t*(189) = -.89, *p* = .38
**TAU Group**					
Hope	6.35 (1.39)	6.52 (1.34)	6.66 (1.44)	6.52 (1.30)	
Meaning in Life	3.62 (.68)	3.68 (.55)	3.76 (.62)	3.64 (.89)	
Mastery	3.51(.78)	3.56 (.85)	3.68 (.88)	3.70 (.90)	
Self-Esteem	2.72 (.45)	2.77 (.36)	2.82 (.44)	2.80 (.41)	
Depression	.27 (.24)	.27 (.24)	.-26 (.24)	.26(.25)	
Life Satisfaction	.73 (.16)	.74 (.15)	.75 (.16)	.73 (.18)	
Stroke Knowledge	3.46 (2.41)	5.45 (1.90)	5.76 (1.93)	5.86 (2.17)	

Table 3: Comparisons of Outcome Measures in Intervention & Control Groups across T0 to T3 (N=192).

**Selected Measures Tested**	**Time effects**	**Pairwise Comparisons**		
		T_0-T_1	T_0-T_2	T_0-T_3
Hope				
Intervention	F (3, 219) = 7.94, p < .001***	p = .01**	p <.001***	p =.04*
TAU	F (3, 237) = 3.15, p = .03*	ns	p =.06	ns
Meaning in Life				
Intervention	F (3, 219) = 12.86, p < .001***	p <.001***	p <.001***	p <.001***
TAU	F (3, 246) = 3.28, p = .02*	P = .07	ns	ns
Mastery				
Intervention	F (3, 210) = 4.38, p < .001***	ns	p =.02*	p =.03*
TAU	F (3, 243) = .81, p = .49	ns	ns	ns
Self-esteem				
Intervention	F (3, 216) = 4.38, p < .001***	p < .001***	p < .001***	p < .001***
TAU	F (3, 243) = 2.70, p = .49	ns	ns	ns
Depression				
Intervention	F (3, 222) = 5.91, p = .01**	ns	ns	**p = .05***
TAU	F (3, 243) = .18, p = .91	ns	ns	ns
Life Satisfaction				
Intervention	F (3, 222) = 4.73, p = .01**	ns	p =.01**	p =.01**
TAU	F (3, 243) = .86, p = .46	ns	ns	ns
Stroke Knowledge				
Intervention	F (3, 219) = 30.63, p < .001***	p < .001***	p <.001***	p <.001***
TAU	F (3, 243) = 43.35, p < .001***	p <.001***	p <.001***	p <.001***

*Note. *p < 0.05. **p <0 .01. ***p <0 .001.*

Table 4: Mean Differences between Intervention and TAU group by Outcome Measures between T_0 and T_1, T_0 and T_2 between T_0 and T_3 (*N*=192)

Outcome Measures Tested	Mean differences between intervention and TAU group (*p-value*)		
	T_1-T_0	T_2-T_0	T_3-T_0
Hope	.07 (.71)	.28 (.05)*	.25 (.04)*
Meaning in Life	.10 (.28)	.26 (.04)**	.28 (.02)**
Mastery	.14 (.26)	.17 (.05)*	.13 (.01)**
Self-Esteem	.18 (.24)	.28 (.06)	.25 (.05)*
Depression	-0.003 (.93)	-.02 (.40)	-0.15 (.04)*
Life Satisfaction	.01 (.61)	.24 (.03)*	.21 (.05)*
Stroke Knowledge	.11 (.73)	.16 (.67)	.32 (.43)

**p* <0 .05. ***p* <0 .01. ****p* <0 .001.*

Table S7 synthesis stage 3 mind mapping

Outcomes from the intervention	Suggested mechanism which explains positive outcomes
**Introducing of the concept of hope and increasing positivity about the future**.	Social comparison<br><br>Speaking with other stroke survivors<br><br>Take time to listen.<br><br>Listening to recovery stories of other stroke survivors<br><br>Platform to sharing stories. similar experiences and situation<br><br>Felt connected.<br><br>Relatedness validation of the feeling of suffering (affirmation support)

	Social control<br><br>The group context created a space for individuals to share and listen to other stories, as well as to observe how other improved and successfully managed difficult situations after their stroke.<br><br>Using humour
**Increasing motivation and creating inspiration**	learning from others and sharing various experiences.<br><br>Meeting other stroke survivors who had positive experiences after stroke.<br><br>Social comparison :Observing how other stroke survivors succeeded after their stroke was motivating.<br>Social upward comparison<br><br>Peer pressure<br><br>Interacting with peer supporter, inspired new stroke survivors to purse a new role in the future.
**Impact on social well-being**<br>**Decreasing sense of loneliness after stroke**	Having time to share, listen and relate to others who had gone through similar experiences .<br><br>Similarity in circumstances decrease sense of loneliness.<br><br>sharing concern and problems created a sense of relatedness and validated suffering.<br><br>Meeting and interview with other stoke survivor was something to occupy their day after stroke.

**Developing valuable relationship and increased social interaction**	The group context created an opportunity to make new friends in common to whom stroke survivors could relate.<br><br>Sense of community that was created in the group could led to develop a long-term valuable relationships , support and network for participants and continued validating the sense suffering outside the group context .
**Impact on emotions**<br><br>**Impact on depression**<br><br><br><br><br><br><br><br><br><br>**Impact on fear**<br><br><br><br>**Related to Negative emotions :**	Sharing experiences with other stroke survivors and validating own feelings and experiences<br><br>Being occupied by attending the peer group.<br><br>Being in a group of other stroke survivors who had disabilities, yet.<br>showed positive attitudes .<br><br>Ability to express negative emotions.<br><br>Peer support<br><br>Related to positive outcome :<br><br>Taking time to listen , sharing stories with peers and validating suffering.<br>Understating their stroke assisted in decreasing sense of fear.<br><br>For stroke survivors :Sharing experiences of stroke.<br>Recalling distressing memories<br><br>For peers who provide intervention :<br>Reliving the distress related to earlier experiences of stroke.
**Enhanced ability to cope and recover**	Sharing stories with others who had successful experiences of recovery.<br><br>Group context created a platform where stroke survivors could provide advice on coping after stroke .<br><br>A peer motivated an individual to practice self-management skills.

	The dialogue approachenhanced ability to copy by ;1)explaining their coping problems ;2)clarifying their coping choices; 3)encouraging them as they sought various coping approaches and 4)assisted them as explored unexpected situation (9)
**Providing informational support**<br>**Gaining knowledge and information about stroke, how to recovery from stroke and locating and accessing stroke support services and opportunities in the community**	**Group context** created a platform to ;(1) to shar and listen to other people's experiences and (2) to interact with stroke survivors at various stages of recovery .

Table S8 synthesis stage 4 the thematic development

Theme	Subtheme	Code	Unite
**Outcomes from the intervention**	**Introducing the concept of hope after stork and helped stroke survivors to be positive regarding the future**.	**Definition of subthemes::**<br>**Discussion around how hope was introduced to their lives after the incident of stroke and how participants learned to be positive regarding future .These positive outcomes occurred through several mechanisms which are following;**<br>(1) Talking to someone who had gone through a similar situation and taking time to listen and shar stories of others was identified as validating the feeling of suffering (affirmation support) (1)<br>(2) Being in a group with other stroke survivors who were in a similar situation and observing how they improved helped participant to learn to be positive regarding the future (2).<br>(3) The narrative aspect of the intervention (Listening to the recovery stories of other stroke survivors could led to the realisation that a person after	

		stroke could recover which as consequence installed hope that did not exist previously (10).<br><br>(4) Being in a group with people with similar situation could led to unite the experiences (validate the suffering) (10)<br><br>that emotional support was provided during the initial in- hospital visit in the form of hope, encouragement and re-assurance .Emotional and affirmational support occurred through taking time to listen, sharing of stories and validation of feelings(1)<br><br>:I honestly didn't want them to leave because I just wanted to continue talking to someone who actually had gone through what I went through. (New Stroke Survivor-Sylvie, initial interview) (1)<br><br>I've learnt to stay **positive** by seeing people who were in similar situations who are now a lot better... being with another who had had a stroke was encouraging as she was in my situation... **I feel more positive for the future**..... If I hadn't have attended the group I wouldn't be as **positive as I am now**." [Patient 7]) (2)<br><br>Eleven participants, 5 recipients and six peer support-, agreed with the item " things seem more **hopeful since joining the group**<br><br>Seventeen participants, all except one of the peer supporters, agreed with the item " group helps me feel more positive about my future". (2)<br><br>Just by going and listening to their [other survivors] stories…there is really something to that. Learning the stories of recovery … helped me [learn] people can recover. It gave me hope where I thought through the years that you couldn't recover." –Irene, 5 years post-stroke (10)	

		We're not alone. Everybody else is the same as us. I've learned from coming [to the group] that there is life after stroke." –Tom, 7 years post-stroke (10)<br><br>There may be limits to the physical comeback that you can make but as far as keeping yourself positive, which is a constant battle … this group and other avenues too: church, your other relationships with people …That's the biggest thing on keeping your mental positive attitude going, and in the final analysis that's almost everything." (7)<br><br>Viewing the video narratives provided some **hope** and less fear to overcome stroke challenges.<br><br>The video was an aid to their plight that there was life after stroke.(13)<br><br>They had a **positive** outlook towards stroke recovery and were willing to do better to improve their health condition.(13)<br><br>I feel that I have to follow the advice, for example, taking medicine, doing blood test… that have been mentioned… (The videos) seem to **inspire** us to take care of health so that we won't get sick. Perhaps to give encouragement makes me feel that I can recover from stroke if follow all the advice [P5] (13)<br>Usually, if you never had a stroke before, you don't really care about watching the videos. Once you had (a stroke), you'll realize that… health is important… you have to take care of it… watch their story… that's it! [P1]<br>Now I ask my doctor more questions if I don't understand…. [P2](13)<br><br>The noted change patterns over time were further investigated in their mean plots, which suggested that the patterns of change over time in the outcome measures across the intervention and the TAU groups were statistically different. Further, many of these positive effects were sustained at 4 months post intervention, in the	

		intervention. Pairwise comparisons revealed that hope, meaning in life, self-esteem and stroke knowledge had significant changes between T 0 and T 1 , T 0 and T2, and T 0 and T 3 (Table 3) (6)<br><br>**Intervention group**<br><br>T0-T1: p = .01**<br><br>T0-T2:p <.001***<br><br>T0-T3p =.04* (6)<br><br>**Control group:**<br><br>T0-T1 :ns<br><br>T0-T2 p =.06<br><br>T0-T3: ns (6)<br><br>NT shows a higher improvement in most outcome scores over the conventional psycho-education intervention. Results further indicate that the intervention group significantly improved hope (R2=0.04, F (1, 176)=7.24**, p = .05),** (6)	
	**Increasing motivation and creating inspiration**	**Definition: Discussion around how stroke survivors were encouraged and empowered by their peers. Motivation was increased through the following mechanisms.**	

		(1) Motivation from peers: learning from others and sharing various experiences. (3)<br>(2) Observing how other stroke survivors succeeded after the incident of stroke was motivating. (3)<br>(3) Being in a group with other stroke survivors and observing how they improved helped participant to learn to be motivated to do better (3)<br>(4) Meeting other stroke survivors who had positive experiences of recovery was motivating to new stroke survivors (1)<br>(5) interacting with peer supporter, inspired new stroke survivors to purse a new role in the future (as peer supporters) (1)<br><br>Motivation from peers was mentioned by a number participants. It was spoken about in relation to vicarious experience or learning from others. Seeing others succeed could be particularly motivating: (3)<br><br>Sometimes it is peer pressure. You see someone doing well and you want to get better as well so it spurs you on a bit. (Paul) (3)<br><br>That emotional support was provided during the initial in- hospital visit in the form of hope form of hope and encouragement (1)<br><br>The visit from the peer supporters encouraged and motivated the stroke survivors to work towards recovery (1)<br><br>Peer supporters were also seen as a source of inspiration (1)<br><br>some stroke survivors who expressed an interest in pursuing a peer supporter role in the future.(1)<br><br>Sixteen participants, all except one recipient and one carer, agreed with the item "	

		is group inspires me about the future". (2)<br><br>Social upward comparison • SGm: 'Because of realizing that Mister IKTm is better than me I want to get there also' (12)<br><br>The motivational cues inspired the patients and raised confidence among themselves. … (sharing) someone's experience to change others'mind. Sometimes, we need to listen to their stories for us to make a change [P4] (13)	
	**Impact on social well-being Decreasing sense of loneliness after stroke and developing valuable relationships and increased social interaction**	**Definition: discussion around how the intervention of interest had positive outcomes on social well-being which were the following: (1) decreasing the sense of loneliness following stroke, (2) developing valuable relationship (friendship relationships) and (3) developing long-term social relationships and support which occurred via the following mechanism.**<br><br>• Having time to share, listen and relate to others who had gone through similar experiences decreased the sense of loneliness (1)<br>• Similarity in circumstances decrease sense of **loneliness** (2)<br>• sharing concern and problems created a sense of relatedness , validated suffering and minimised the sense of **loneliness** (7)<br>• Talking to other stroke survivors and sharing similar experiences was identified as validating the suffering and decrease sense of l**oneliness** (3)<br>• Meeting with other stoke survivor was something to occupy their day after stroke (4)<br><br>• The group context created an opportunity to make new friends in common to whom stroke survivors could relate (2)<br>• Being in a peer support group which based on the group dynamic theory and the social learning theory facilitated opportunities to makes new	

		friendships and increased social interaction inside and outside the group context with people like minded (5)<br>• Sense of community that was created in the group could led to develop a lifelong valuable relationships , support and network outside of the group context that continued validating the sense suffering outside the group context .(7)<br><br>• Stroke survivors in the group had become like friends because they cared and took time to listen , in contract to other people (10)<br>•<br>**<u>Related to decreased loneliness.</u>**<br><br>After having someone who had gone through a similar experience take time, listen, share experiences, and make a connection, stroke survivors reported feeling validated and less alone(1)<br><br>It made me realise I was not alone, that there are many others in the same position.” [Patient peer supporter 2]. (2)<br><br>shared experience may reduce feelings of loneliness as Paul illustrates (3)<br><br>That is good if you know there are others in the same position as you. Going, going through the same thing as you. So you don’t think you are the only one... It’s good talking to other stroke survivors because with their experiences you know you are not alone then. Because it is very demoralising once you have had your stroke. (Paul) (3)<br><br>-I think partly it was like a day out for her, breaking the monotony, but once her family came back she had something to fill the day. When I had my stroke and was looking for help, anything I was offered I would go for it. For me I was looking for solutions to problems, I was meeting with her to work on solving problems. Her problem was loneliness, so I was the solution. (Peer 2) (4)	

		Participant R2G3 said, “It makes you feel like you are not alone. Like there is someone else out there that is having the same problems and concerns that you do. And how they are dealing with it makes a difference(7)<br><br>-I think that if I had’ t had this course<br>[intervention], I would have felt terribly alone.<br>(Woman, 66 y.o./group intervention).(9)<br><br>Peer support groups helped survivors not feel alone or isolated. Almost all participants commented on the importance of connecting with other survivors. At least half of the survivors expressed gratitude to have received either a visit in the hospital or a phone call from a stroke survivor. They articulated that the isolation of their stroke seemed somewhat alleviated once they connected with others in a similar situation. “You find you’re not alone. [The group] is an extended family who knows what you’re going through.” –Angela, 11 years post-stroke (10)<br><br>You don’t feel quite so much out on a limb. When you’ve heard [that] other people have gone through the same procedures you had.” **[Patient 6].** (2)<br><br>“It made me realise I was not alone, that there are many others in the same position.” [**Patient peer supporter 2].** (2)<br><br>**<u>Related to developing valuable relationships and increased social interaction</u>**<br><br>it the group has changed my life as I have made friends with other people who have had a stroke.” [Patient 7].(2)<br><br>Member response to post participations survey :5 out 13 reported “strongly agree “ that the program helped them to make new friends and 5 out of 13 reported” agree” (5)	

		Descriptive data about social and communication activity outside of the group were tracked on the post group survey. For example, members were asked if they met with anyone socially outside of the group or if they used communication path- ways such as Facebook or texting to socialize/communicate outside of the group. Approximately, half of the individuals (*n* = 6) reported interaction with another member outside of the group context in various formats including text (*n* = 2), Facebook (*n* = 2), met or attempted to meet outside of the group (*n* = 8), email (*n* = 5), and talk on the phone (*n* = 6). (5)<br><br>Equally, over half of the members (*n* = 8) reported socialization being their favourite aspects of the group process .example, members reported new friendships and social networking were most beneficial. (5)<br><br>The social domain<br><br>The CIQ social domain did not reach significant:<br>Mean pre score: 8.23<br>Mean post score: 9.08 (5)<br><br>(*p* value 0.148) (5)<br><br>The sense of community was unique to the support group experience, and impacted participants' lives beyond scheduled stroke support group meetings. Participant R3G2 noted, "I think this group has become more than a support group once per month. They are almost like family (7)<br><br>… They have become friends and support outside of the meetings." Additionally, participant R2G1 stated, "We love the relationships that we are building with other people. Even if we are in a store ... it's like, 'oh they are in our stroke group.' It is important that outside of this room even, it reminds you that there are other people experiencing the same reconfiguration of their life that we are." (7)<br><br>They [other survivors] speak to me and then they'll listen. Some people would say 'oh, I can't be bothered'. They're like friends because they're always willing to help you'" –Lillian, 1.5 years post stroke (10)<br><br>Negative case :	

		The peers identified that although they had not experienced this difficulty, they could see how there was the potential for the stroke survivor to become attached to the peer: It's hard to meet somebody one to one. To meet them at a personal level and then suddenly, that is it, you are never going to see them again. This could be a problem because if you get the wrong sort of people, the ones that cling on all the time they would be phoning you all the time just for a bit of company. (Peer 1) (4)<br><br>**The facilitators gained a better understanding of the participants as individuals because of sharing stories with the groups**<br><br>As part of the evaluation, the facilitators were asked.<br>to answer the following question by the trainee clinical<br>psychologist: 'How was the Share **Your Story**<br>**Group useful for you as therapi**st?' Responses were<br>written down verbatim and grouped. This resulted in<br>the following categories: (8)<br><br>■ Getting to know patients as people (six responses (8)	
	**Impact on emotions**<br><br>**Depression**	**Definition: discussion around the impact of the intervention of interest on depression and related emotions**<br><br>**Qualitative result related to depression outcome :**<br><br>**Mechanism related to depression outcomes** :sharing experience with other stroke survivors and validating own suffering helped in reducing depression. (7) (10)<br><br>Many participants shared feelings of frustration, depression, and isolation. These feelings stemmed from fear, lack of strength, and feeling overwhelmed by the challenges of adjusting to a new normal after stroke. However, sharing their experiences in peer stroke support groups was helpful. Participant R2G1 noted, "I felt that I was a little depressed before I came … I found that being around other	

		people that's gone through the same thing helps.**"(7)**<br><br>**Mechanism related to depression outcomes.**<br><br>1. Being in a group of people with similar condition and who had disabilities ,yet had positive attitudes could led to increase spirit and decreased depression (10)<br>2. Discussion and talking with other stoke survivors and express emotion (negative) lead to validate suffering/ not alone feeling this way+ support =decrease phycological distress (depression) (10)<br>3. attending the peer support group=lead to occupy patients with something to do every month=lead to elevate the depression (10)<br><br>Although many survivors experience depression post-stroke, their support group played a role in overcoming depression (10)<br><br>Of those who experienced stroke, the majority expressed their support group played a significant role in overcoming depression. (10)<br><br>Only 1 participant did not report depression was a part of their stroke experience. (10)<br><br>"Being together with such a happy group who had disabilities too helped me keep my spirits up so I don't let myself get down." –Lena, 2.5 years post-stroke (10)<br><br>"I personally thought about suicide. It's scary. In forums they [survivors] all said we felt depressed…in the beginning it's very normal. I'm not alone feeling that way." – Angela, 11 years post-stroke (10)<br><br>They [the support group] helped a lot. I talked about depression and why I wanted to commit suicide and they were really supportive." –Tom, 7 years post-stroke (10)	

When I first came home from the hospital I wanted to go home, sit in a corner and cry…..now every month I have something to go to.” –Aryln, 7 years post-stroke (10)

Quantitative results regarding depression :

Intervention group: T0(baseline),*M*(SD).32(.25);T1(interim) .33(.27);T2(post intervention),-.25(.26) and T3(4 months)-.20(.26) (6)

Table 2: Means, Standard Deviations and Baseline Differences between Groups of Outcome Variables Over Time (*N*=192).

Variable Tested	T_0	T_1	T_2	T_3	Baseline Difference Between Groups
**Intervention Group**	*M* (SD)	*M* (SD)	*M* (SD)	*M* (SD)	*t*-test
Hope	6.13 (1.23)	6.62 (1.35)	6.89 (1.28)	6.75(1.58)	*t*(189) = -1.14, *p* = .25
Meaning in Life	3.47 (.65)	3.77 (.62)	3.93 (.59)	4.33 (.71)	*t*(189) = -1.73, *p* = .86
Mastery	3.39 (.85)	3.60 (.77)	3.88 (.86)	3.85 (.77)	*t*(188) = -1.18, *p* = .24
Self-Esteem	2.60 (.44)	2.85 (.44)	2.99 (.32)	2.94 (.48)	*t*(189) = -1.57, *p* = .12
**Depression**	**.32 (.25)**	**.33 (.27)**	**-.25 (.26)**	**-.20(.26)**	***t*(190) = 1.44, *p* = .15**
Life Satisfaction	.71 (.19)	.78 (.17)	.88 (.15)	.84 (23)	*t*(190) = -.62, *p* = .54
Stroke Knowledge	3.26 (2.15)	5.05 (1.88)	5.49 (2.09)	5.54 (2.17)	*t*(189) = -.89, *p* = .38
**TAU Group**					
Hope	6.35 (1.39)	6.52 (1.34)	6.66 (1.44)	6.52 (1.30)	
Meaning in Life	3.62 (.68)	3.68 (.55)	3.76 (.62)	3.64 (.89)	
Mastery	3.51(.78)	3.56 (.85)	3.68 (.88)	3.70 (.90)	
Self-Esteem	2.72 (.45)	2.77 (.36)	2.82 (.44)	2.80 (.41)	
Depression	.27 (.24)	.27 (.24)	-.26 (.24)	.26(.25)	
Life Satisfaction	.73 (.16)	.74 (.15)	.75 (.16)	.73 (.18)	
Stroke Knowledge	3.46 (2.41)	5.45 (1.90)	5.76 (1.93)	5.86 (2.17)	

Table 3: Comparisons of Outcome Measures in Intervention & Control Groups across T0 to T3 (N=192).

Selected Measures Tested	Time effects	Pairwise Comparisons		
		T_0-T_1	T_0-T_2	T_0-T_3
Hope				
Intervention	F (3, 219) = 7.94, p < .001***	p = .01**	p <.001***	p =.04*
TAU	F (3, 237) = 3.15, p = .03*	ns	p =.06	ns
Meaning in Life				
Intervention	F (3, 219) = 12.86, p < .001***	p <.001***	p <.001***	p <.001***
TAU	F (3, 246) = 3.28, p = .02*	P = .07	ns	ns
Mastery				
Intervention	F (3, 210) = 4.38, p < .001***	ns	p =.02*	p =.03*
TAU	F (3, 243) = .81, p = .49	ns	ns	ns
Self-esteem				
Intervention	F (3, 216) = 4.38, p < .001***	p < .001***	p < .001***	p < .001***
TAU	F (3, 243) = 2.70, p = .49	ns	ns	ns
Depression				
Intervention	F (3, 222) = 5.91, p = .01**	ns	ns	**p = .05***
TAU	F (3, 243) = .18, p = .91	ns	ns	ns
Life Satisfaction				
Intervention	F (3, 222) = 4.73, p = .01**	ns	p =.01**	p =.01**
TAU	F (3, 243) = .86, p = .46	ns	ns	ns
Stroke Knowledge				
Intervention	F (3, 219) = 30.63, p < .001***	p < .001***	p <.001***	p <.001***
TAU	F (3, 243) = 43.35, p < .001***	p <.001***	p <.001***	p <.001***

*Note. *p < 0.05. **p <0 .01. ***p <0 .001.*

A reduction in the mean, for both anxiety and depression scores was found.

Mean pre group depression approximate just at 8.5 of Hospital Anxiety and Depression Scale (HADS) and Mean of post group depression of HADS approximately just above 7.5 (8)

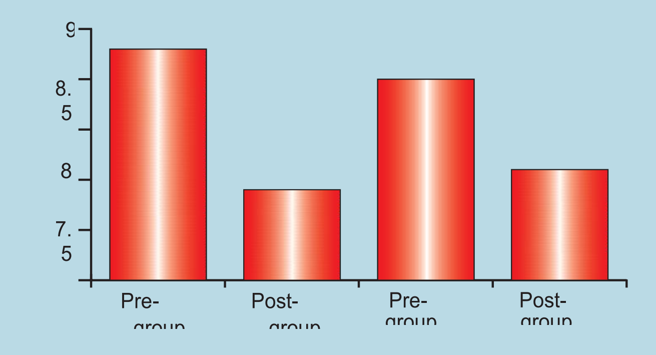

Figure 1. Hospital Anxiety and Depression Scale mean scores pre- and post-group

, but T-test results showed no significant changes in HADS scores before and after the group (n=34) (8)

Affective mood states, measured by the VAMS at the end of the first and the last BNI also changed, as shown in table 4. While **little improvement was demonstrated for the mood state 'happy'** with an increase in the mean T-score from 40.85 to 47.40 (Cohen's d = .25), the mood states 'confused' and 'tense' decreased moderately from 52.96 to 46.23 (Cohen's d = .56) and from 53.52 to 46.50 (Cohen's d = .45), respectively. For all other mood states there were expected trends towards a growth of positive emotions and a reduction of negative emotions. We also conducted **the VAMS at the end of the first and the last GI. Again we found expected trends. The mood state 'tired' decreased from 49.00 to 43.33 (Cohen's d = .30) (12**)

Table 4. Values for mood states VAMS (Stern 1997) at end of the first and the last biographic–narrative interviews (BNI) and group interventions (GI) (12)

Test	Baseline (end BNI 1) (*n* =27), mean *T*-scores (95% CI)b	Post (end BNI 5) (*n* = 26), mean *T*-scores (95% CI)	Baseline versus post-test *P*-value (outcome)a	Cohen's *d*
VAMS afraid	47.41 (2.61)	46.62 (1.68)	.50	.12
VAMS confused	52.96 (5.77)	46.23 (2.12)	.01	.56
VAMS sad	52.37 (6.73)	47.81 (3.25)	.08	.30
VAMS angry	50.04 (5.10)	47.08 (3.39)	.25	.21
VAMS energetic	44.37 (5.11)	48.46 (5.02)	.27	.14
VAMS tired	46.96 (4.00)	46.92 (4.22)	.78	−.03
VAMS happy	40.85 (5.48)	47.40 (4.52)	.01	.25
VAMS tense	53.52 (4.42)	46.50 (2.97)	.00	.45
	**Baseline (end GI 1) (*n* = 25), mean *T*-scores (95% CI)**	**Post (end GI 7) (*n* = 24), mean *T*-scores (95% CI)**		
VAMS afraid	47.33 (4.73)	46.86 (3.27)	.40	.36
VAMS confused	48.00 (5.48)	45.35 (2.03)	.40	.58
VAMS sad	52.96 (8.85)	47.76 (5.24)	.32	.38
VAMS angry	48.75 (6.54)	45.19 (2.15)	.37	.41
VAMS energetic	45.25 (6.03)	48.62 (15.17)	**.48**	−.09
**VAMS tired**	**49.00 (6.99)**	**43.33 (11.70)**	**.00**	.30
VAMS happy	42.88 (6.92)	45.52 (14.92)	**.23**	−.02
VAMS tense	51.21 (4.86)	49.86 (11.80)	.93	.18

Notes: [a]Repeated measures *t*-test, two-tailed.
[b] *n* varies because not all participants attended at each session.

TABLE 4
Values for mood states VAMS (Stern, 1997) at end of first and last biographic-narrative interview (BNI) and group intervention (GI) (*n* varies because not all participants attended at each session) (11)

	Baseline (end BNI 1) (n = 17) Mean -t-scores (95% CI)	*Post (end BNI 5); (n = 16) Mean t-scores (95%CI)*

			Baseline vs.Post-test p-values¹ (outcome)
**VAMS** afraid	48.06 (4.32)	46.38 (2.35)	.30
VAMS confused	54.88 (8.99)	46.50 (3.65)	.03*
VAMS said	52.88 (10.33)	48.13 (5.33)	.12
VAMS angry	52.71 (8.42)	47.44 (5.49)	.20
VAMS energetic	44.35 (7.33)	49.00 (7.11)	.39
VAMS tired	48.65 (6.12)	46.38 (5.47)	.64
VAMS happy	39.00 (7.66)	46.53 (7.13)	.02*
VAMS tense	54.47 (6.59)	46.63 (4.49)	.01**

Test	*Baseline (end GI 1); (n = 14) Mean t-scores (95% CI)*	*Post (end GI 7) (n = 13) Mean t-scores (95% CI)*	*Baseline vs. Post-test p-values¹ (outcome)*
**VAMS** afraid	48.00 (4.44)	46.00 (2.70)	.56
VAMS confused	49.43 (5.48)	45.00 (1.53)	.30
VAMS said	51.64 (8.85)	46.23 (3.37)	.42
VAMS angry	49.00 (6.54)	44.69 (1.55)	1.00
VAMS energetic	44.93 (6.03)	47.46 (8.09)	**.86**
VAMS tired	52.07 (6.99)	45.15 (6.61)	**06trend**
VAMS happy	42.36 (6.92)	43.92 (8.25)	**.46**
VAMS tense	49.79 (4.86)	46.23 (5.67)	**.44**

[1]Repeated measures *t*-test, two-tailed.

Definition :discussion around how the intervention of interest seemed to reduce the sense of fear and instilling a feeling of reassurance

Qualitative result :

Viewing the video narratives provided some hope and **less fear to overcome stroke challenges. I**t's a bit of both worrying and confidence… There is always a worry about what can happen, but it also gives you an idea (on) what to do, and what to be careful, and what to be aware [P3] (13).

	Fear	**Taking time to listen , sharing stories with peers and validating the suffering felt during the hospitalisation period offered emotional support in the form of reassurance.**<br><br>That emotional support was provided during the initial in- hospital visit in the re-assurance(1)<br><br>**Fear was commonly reported by participants and peer support groups appeared to help to understand the condition, which assisted in overcoming the fear after stroke.**<br><br>far was emphasized as a great challenge in accepting stroke..<br>[10]<br><br>When I first had the stroke, I went to my family doctor 3-4 times a week thinking I would be having a stroke. I was always scared," and indicated "**the group helped me overcome my fear." –**Kelly, 4 years post- (10)<br><br>"Don't be so fearful after you have [had your stroke]. I remember being afraid and that's **where the group helped**." Irene, 5 years post-stroke (10)<br><br>**Negative case** :There may conversely be some negative impacts of the interventions on emotions of both stroke survivors and peer supporters which might occurred through the following mechanism :<br><br>• Some aspects of the intervention such as sharing experiences of stroke and recalling distressing memories might led to arise intensive feelings (3).<br>• Hearing and talking about stroke could be "distressing" and "difficult" (3)<br>• Sharing experiences of stroke within a group context might impact negatively on physical and emotions health (increased stress) (3)<br>• The nature of the group (a place where both negative or positive emotions were expressed could be emotionally challenging to some individuals (3).	

		Ability to cope emotionally – "perhaps not everyone can cope" The ability of each individual to cope with the potential emotional demands of a group SMP was discussed by participants. Some aspects of a group may be emotive for some individual's such as, **discussing experiences of stroke and remembering things that are distressing. (**3)<br><br><br>Five stroke survivors spoke about how they would cope emotionally when hearing and talking about stroke. Thomas reflects on times he has had to hear about stroke, such as in the paper. He says he does not like it as he finds it both "**difficult**" and "**distressing"** to hear(3)<br><br>James said he would not want to put himself in a situation in which he might get **stressed as he worries** it would aggravate his atrial fibrillation (3)<br><br>Margaret, whilst more certain about how she might react emotionally, also sees the experience **as challenging:** (3)<br>"I have found I have got more emotional, I will cry at the drop of a hat, you **know, happy times,** or **sad times.** And erm, if I had somebody who was in a group who was reduced to tears I would be too. .**... it is not a nice feeling I suppose**." (3)<br><br>The concern that all these participants share is summarized by Ben, who talks about emotional management in relation to group programs:<br><br>I suppose the danger with rolling it out to everyone, is perhaps not **everyone can cope**. **Because it's not very good news this stuff you are being told. (3)**<br><br>Negative case related peers who provided the intervention : there might be a potential emotional impact of the intention on peers as the intervention might trigger experience relates to their initial stage of stroke.<br><br>While concern was expressed by program coordinators about the potential emotional impact on peer supporters for whom the visit may trigger past feelings	

		related to the initial experience of stroke, none of the peer supporters interviewed described experiencing these types of feelings (1)<br><br>**Quantitative result :**<br><br>**VAMS** afraid (P=.056) (11)<br>**VAMS** afraid (p= 0.36) (12)	
	**enhanced ability to cope and recover**	**Definition : discussion around how the group context enhanced ability to cope and recovery after stroke which occurred through the following mechanisms.**<br>• Through **sharing experiences** and how other stroke survivors **coped** after stroke , an individual picked up ideas on how cope with their own situation (10)<br>• Humour and positivity in groups were also frequently mentioned as important components of effective recovery (10).<br><br>• Through **hearing how other stroke survivors coped** with their challenges , participant discovered new ways to **cope** with their own difficulties and were able to transfer these experiences and implement them to their day to day lives (action planning) (2)<br>• **Through creating a platform where stroke survivors could provide advise o**n coping after stroke (2)<br>• **A peer motivated** an individual to practice **self-management skills** by setting a goal ,breaking it down into manageable measures and working towards which had a positive effect on his confidence (4)<br>• **The dialogue-based intervention** in both formats (group and individual) according to participants assisted them in cope with their difficulties after stroke. Their difficulties ranged from carrying out daily task and solving practical issues to comprehending and coping with their own emotional response as well as those of families, friend, and co-workers (9**) .This**	

		**occurred through the following .Mechanism;**1)explaining their coping problems ;2)clarifying their coping choices; 3)encouraging them as they sought various coping approaches and 4)assisted them as explored unexpected situation (9)<br><br>• By Listening to how other stroke survivors **handled** their situation, , they learned new to address various circumstances (9)<br><br>"When they [other survivors] talked about their experiences and how they coped…I learned tips on how to get on with it." –Lena, 2.5 years post-stroke (10)<br><br>Although many of the participants expressed gratefulness for the contributions their support group had brought to their lives and recovery (10)<br><br>Laughter and being positive were also commonly raised as key components of a successful recovery and of strong peer support groups (10)<br><br>Fourteen participants, eight recipients and six peer 2supporters, agreed with the item "When I hear how other people are coping with their problems in the group, I often learn new ways to cope with my difficulties".(2)<br><br>13 participants, eight recipients and five peer supporters, agreed with the item "I pay attention to how others handle difficult situations in my group so I can apply these strategies in my own life (2)<br><br>fifteen of the participants, eight recipients and 7 peer supporters, also agreed with the statement "In the group, other members advise me what I should do about difficult situations and life decisions(2)	

		Nine participants, four recipients and 5 peer supporters, agreed with the statement "In the group I get 'how to on improving my life (2)<br><br>One participant further explained how he was taken out of his comfort zone and was encouraged to identify an initial goal and work towards it by breaking it down into necessary steps. Although challenging, the participant could see the need for this. In his words: (4)<br><br>So, I think [Peer] was … more goal-orientated than me. I think for me, it was useful to have that structure […] so … it was useful that he was having those. (P4) (4)<br><br>The participant and peer agreed on a goal which involved the participant being able to take a digital photograph and follow the necessary steps to save it, download it, edit it and finally print it.The participant expressed how he could see the change as the coaching progressed: But as I engaged and went on through the weeks … I know that I was able to …, I could absorb the information and … be able to apply it. (P4) (4)<br><br>By Session 6 the participant was able to complete the photographic process and he expressed this had a positive impact on his confidence (4)<br><br>**Being supported in their attempts to cope with the situation**<br>The participants struggled to cope with their new and unknown situations after the stroke. The issues they struggled with varied widely, from performing daily activities and solving practical problems to understanding and coming to terms with their own emotional reactions and those of their family, friends and colleagues. Facing different social situations within and beyond their family entailed many challenges. (9)<br><br>The participants reported that the intervention helped them cope with their struggles. Participants in both the individual and group-based interventions emphasised that the dialogues helped them cope by clarifying what their coping challenges entailed, illuminating their coping options, supporting them as they tried	

		different oping strategies and supporting them as they analysed unexpected situations (9)<br><br>When a traumatic event occurs, such as a stroke , **it is important to be able to share experiences with those** that had been in similar situations (9)<br><br>The participants in the group-based intervention also reported that by listening to how other stroke survivors managed their situation, they learned new ways to approach different situations: P: I always left [the meetings] a little inspired! I think it is important when a serious thing like a stroke happens, that one may exchange experiences with others who have been in the same situation… . That is what has been most important for me – to be together with people in the same situation. (9)<br><br>**Main theme: Informative and reminder • The videos narratives were a "trigger" toward proactivity and enhanced patients' awareness about stroke and its preventative treatment**. (13)<br>They remind us of important medicine… They remind us of the danger of the second stroke… to take medicine well and to have a healthy lifestyle [P7] Helpful….more understanding about stroke [P6]<br>Awareness… before that we were not really concerned about our health. Now, after the advice it's different... like a guide [P4]<br>Patients can recover from stroke and (it) won't recur if we take the medicine prescribed by doctors according to the right schedule on time [P2] (13)<br><br>Ten of the 13 members reported "agree" or "strongly agree" they were able to identify a new coping strategy, and eight of the 13 members reported they actively used the strategy outside the group. (5)<br><br>These findings provide preliminary indication that the YESS group had a positive impact on coping strategies after stroke. (5)<br><br>For example, members were asked if they engaged in volunteer activities, exercise programs, or leisure programs that were presented in the group	

modules. Over half of the members (n = 8) reported they began to engage in various leisure and ADL opportunities outside of the group context. (5)

TABLE 3. Pre and Post group Stroke Impact Scale Domain Scores

Stroke Impact Scale Domain	Median Prescore	Median Postscore	Mean Prescore	Mean Postscore	*p* value
Self-reported recovery score	70 (30–90)	70 (40–95)	62.2	66.2	.196
Strength	50 (25–93.8)	56.3 (25–93.8)	51.9	56.8	.342
Hand function	0 (0–90)	20 (0–100)	28.5	41.2	.138
Mobility	97.2 (52.8–100)	94.4 (55.6–100)	91.7	89.8	.778
ADL/IADL	85 (52.5–100)	90 (44.4–100)	83.2	82.8	.551
Memory	82.1 (57.1–100)	85.7 (60.7–100)	81.9	84	.683
Communication	92.9 (10.7–100)	96.4 (46.4–100)	80	89.8	.214
Emotion	83.3 (50–97.2)	83.3 (44–97.2)	76.9	77.1	1
**Handicap**	59.4 (37.5–100)	75 (53.1–100)	63.5	75.8	**.034***

* Note: $p \leq .05$

TABLE 4. Pre- and Post-group Scores on Community Integration Questionnaire

Domain	Median Prescore	Median Postscore	Mean Prescore	Mean Postscore	*p value*
Total score	14.25 (2–21.5)	19 (13.7–23.75)	14.87	18.22	0.028*
**Home integration**	5 (1–7.5)	5.25 (2.6–10)	4.25	5.99	**0.002***
Social integration	8 (2–12)	9 (6–12)	8.23	9.08	0.148
Productivity	2 (1–6)	3 (1–7)	2.69	3.23	0.334

* Note: $p \leq .05$

TABLE 5. Member Responses to Post participation Survey (*N* = 13)

Answer Options	Strongly Agree	Agree	Neutral	Disagree	Strongly Disagree

I have made new friends	5	5	2	0	1
I provided support to other group members	5	4	3	0	1
**I learned new ways to cope with issues related to stroke**	**3**	**7**	**2**	**0**	1
I am using a coping strategy learned in the group to deal with frustrating or stressful situations	2	6	4	0	1
I know more about community and stroke resources	5	5	3	0	0
I have gained knowledge to advocate for myself	3	7	3	0	0
I feel more hopeful about the future	4	5	4	0	0
I know more about opportunities after stroke (i.e., modified leisure, volunteer, or research opportunities)	4	6	2	0	1
I am more informed about ways to achieve my goals regarding recovery	4	7	2	0	0

Table 2 Groups' Comparison at Various Timelines (14)

		Timeline (Month)			Within Group			Between Groups			(Timeline*Group)		
		T0 (SD)	T2 (SD)	T4 (SD)	F	p	η2	F	p	η2	F	p	η2
MUSE[a]	I	22.97 (5.17)	27.27 (3.25)	27.81 (2.52)	97.00	<0.001**	0.475	**12.41**	0.001**	0.055	42.99	<0.001**	0.167
	C	23.71 (5.44)	24.62 (4.03)	24.42 (4.02)	5.61	0.010*	0.050						
MUSE[b]	I	25.00 (5.85)	27.82 (2.96)	28.38 (3.23)	42.05	<0.001**	0.282	5.10	0.025*	0.023	6.91	0.005*	0.205
	C	24.75 (5.80)	26.29 (4.97)	26.28 (4.88)	14.76	<0.001**	0.121						
MUSE[c]	I	25.61 (5.71)	28.88 (3.43)	28.19 (3.18)	49.11	<0.001**	0.315	7.25	0.008*	0.033	12.22	<0.001**	0.195
	C	25.29 (5.61)	26.39 (5.02)	26.25 (4.73)	8.22	0.003*	0.071						
Sys/BP	I	141.68 (20.21)	134.26 (14.96)	135.71 (13.88)	35.67	<0.001**	0.250	0.35	0.552	0.002	8.537	0.001**	0.038
	C	139.89 (21.31)	137.59 (15.78)	138.11 (15.73)	3.37	0.055	0.031						
SKT	I	7.47 (3.40)	9.34 (3.09)	9.79 (3.11)	137.64	<0.001**	0.563	11.54	0.001**	0.051	32.39	<0.001**	0.131
	C	6.83 (3.59)	7.49 (3.36)	7.81 (3.28)	42.93	<0.001**	0.286						

<table>
<tr>
<td></td>
<td></td>
<td>
<table>
<tr><td></td><td></td><td></td><td></td><td></td><td></td><td></td><td></td><td></td><td></td><td></td><td></td><td></td><td></td></tr>
<tr><td>BIPQ</td><td>I</td><td>52.08 (10.06)</td><td>47.44 (9.29)</td><td>47.79 (8.81)</td><td>53.84</td><td><0.001**</td><td>0.335</td><td>1.73</td><td>0.190</td><td>0.008</td><td>34.16</td><td><0.001**</td><td>0.104</td></tr>
<tr><td></td><td>C</td><td>50.76 (12.12)</td><td>51.08 (11.89)</td><td>51.15 (12.77)</td><td>0.42</td><td>0.594</td><td>0.004</td><td></td><td></td><td></td><td></td><td></td><td></td></tr>
<tr><td>BMQ</td><td>I</td><td>51.46 (6.22)</td><td>48.22 (6.31)</td><td>47.57 (6.15)</td><td>51.39</td><td><0.001**</td><td>0.324</td><td>15.93</td><td><0.001**</td><td>0.069</td><td>71.76</td><td><0.001**</td><td>0.251</td></tr>
<tr><td></td><td>C</td><td>50.91 (6.22)</td><td>52.76 (7.11)</td><td>53.74 (8.00)</td><td>23.82</td><td><0.001**</td><td>0.182</td><td></td><td></td><td></td><td></td><td></td><td></td></tr>
<tr><td>BP/Mon</td><td>I</td><td>2.41 (0.94)</td><td>2.76 (0.98)</td><td>2.87 (1.04)</td><td>27.34</td><td><0.001**</td><td>0.204</td><td>5.23</td><td>0.023*</td><td>0.024</td><td>12.49</td><td><0.001**</td><td>0.055</td></tr>
<tr><td></td><td>C</td><td>2.33 (0.89)</td><td>2.44 (0.91)</td><td>2.44 (0.89)</td><td>5.74</td><td>0.006*</td><td>0.051</td><td></td><td></td><td></td><td></td><td></td><td></td></tr>
</table>
Notes: [a](all medication), [b](antiplatelet), [c](antihypertensive), *The mean difference is significant at p<0.05, **Adjustment for multiple comparisons: Bonferroni p<0.001. Abbreviations: BIPQ, Brief Illness and Perception Questionnaire; BMQ, Belief About Medicine Questionnaire; BP, blood pressure; BP/Mon-BP monitoring; CI, confidence interval; C, control group; I, intervention group; MUSE, medication understanding and use self-efficacy; SKT, stroke knowledge test; Sys/BP-systolic BP; T0, baseline; T2, 6th month; T4, 12th month.
The item of the helpfulness of group “ share your story” on a 10-point Likert-type rating was completed by nearly half of the participants (n=37/80) and the result showed an average rating being 7/10.
The Likert-rating item on helpfulness of the group for the participants’ recovery was completed. by 37 participants. The ratings of helpfulness of the group showed an average of 7 (range 1–10). (8)
</td>
<td></td>
</tr>
<tr>
<td></td>
<td>Providing informational support and enhanced awareness of stroke and increased knowledge</td>
<td>
Definition: discussion around how the intervention of interest provided information support regarding stroke, and how to recover form stroke and how to locate and access stroke support service in the community.
Theme 1: Survivors and caregivers seek and receive knowledge about stroke and stroke recovery from stroke peer support groups. (7)
Pursing and gaining information was the initial reason to participate in the groups. Stroke survivors and their carers needed information about the recovery process , but it was difficult to find .
</td>
<td></td>
</tr>
</table>

		Participants shared that they initially attended stroke peer support groups to learn more about stroke and recovery (7)<br><br>Receiving stroke and recovery related knowledge was important to survivors and caregivers, and difficult to locate. As participant R2G1 stated, “We all go different places and do different things but there is not really a great one source; you just can’t go to the kiosk and say, ‘I would like to have all of these resources for stroke survivors.’ It’s just not there.” (7)<br><br>**One of the reasons that stroke survivors wanted to continue attended the groups was to learn more about their stroke and rehabilitation process**.<br><br>Receiving knowledge about stroke and recovery is one reason participants continued to attend support groups. Participant R5G1 noted, “I want to continue to recover. I don’t want to stop. I want to get stronger each day. I want to get all the knowledge I can possibly get.” (7)<br><br>**Participants gained insight by interacting with other stroke survivors at various stage of rehabilitation.**<br><br>Importantly by meeting with other stroke survivors at different stages of recovery, participants gained knowledge from each other and felt empowered by providing help to their peers. Participant R2G4 said, “Each one of us helped somebody that was having a stroke. If we can do what we do and not be doctors, we can make a big difference right here.” (7)<br><br>**Via group discussion , participants gained a better understanding of any risk associated d with a stoke diagnosis**	

		You discuss things so you can be on the lookout for anything that might be going wrong, or things you need to be looking to get checked out if you are having this symptom or that one that you might not have thought about before(7)<br><br>**Increasing knowledge about stoke not only helped stroke survivors feel more educated but also gave the skills that they could use outside the group context to facilitate recovery process.**<br><br>” In all focus groups, participants reported that the information they received covered multiple stroke-related topics, such as diet, exercise, blood pressure management, stroke prevention and treatment, and stroke research. Having stroke-related knowledge not only made participants feel more informed, but also equipped them with concrete skills they applied at home to further their recovery. As participant R2G2 stated, “We have had different speakers on different subjects … We had a guy who came in and did exercises with us. Of course, we carried that on some at home.” Similarly, participant R1G3 noted, “You discuss things so you can be on the lookout for anything that might be going wrong, or things you need to be looking to get checked out if you are having this symptom or that one that you might not have thought about before.” Professionals such as physicians, therapists, and dietitians were sometimes invited to share their knowledge at support group meetings, often at the request of group members (7)<br><br>Informational support was also offered during the initial and follower up telephone visits. Peer supporters provided written as well as verbal information on resources in the community including those offered by SSAO. (1)<br><br>During the initial visit this information tended to be more general in nature. However, during follow-up telephone calls, information was targeted to the new stroke survivor’s or care partner’s needs and included information on specific services avail- able in the community. Information to assist with both finding and accessing services was provided to six out of the 28 people for whom follow-up diaries were kept (1).	

		**Information about the experiences of coping with stroke provided by peers was valued higher than the information provided by health care professionals.**<br><br>Information received from a peer regarding the experience of living with stroke was generally given more value than that received from a healthcare professional.(1)<br><br>**Peers offered knowledge and information gained from real -life experiences which promoted relatedness.**<br><br>peer supporters] provided sort of reassurance... and provided a real face. When you're dealing with doctors and nurses they're great.... But they're medical people and they can talk to you about what you've just gone through, but the chances are more than likely that they haven't... The people in Stroke Survivors, well they have. (New Stroke Survivor-Mike, initial interview) (1)<br><br>What did these survivors want new survivors to know about stroke peer support groups? (10)<br><br>“They can help you and be very helpful with resources.” – Kelly, 4 years post stroke (10)<br><br>**Via group discussion , participants gained useful information and guidance about after discharge support programs.**<br><br>Several participants felt that peers provided valuable information and advice about post-hospital support services (2)<br><br>I’ve learnt what help you can get when you come home... and how to get it.” [Patient 5].(2)	

		**Stroke survivors who had a lot of experiences since their stroke thought to be the in the best position to support.**<br><br>Individuals with substantial experience subsequent to their stroke were viewed as being in the best position to be helpful. (2)<br><br>"that was the ones that I probably got the most benefit from. the ones that had come in that had had strokes probably a year earlier than I had that were further up the road...and you can ask them questions... what do you do in this situation or another situation?" [Patient 6]. (2)<br><br>**Being in a group with other stroke provided an opportunity to share and hear other people's experiences which led to increased awareness about their condition.**<br><br>The strength of being in a group is that you get to share others' experiences … I had never realised that you could get psychological problems after stroke unless I had seen one of the other participants … I found that very enriching. (Woman, 66y.o./group intervention [9]<br><br>Peer support groups helped survivors understand their stroke (10)<br><br>**Several participants reported that they did not understand their stroke or how to deal with the recovery process at the time of their stroke or during hospitalization period.**<br><br>Many participants indicated that at the time of their stroke and during their hospital stay, they did not feel they adequately understood their stroke or how they could cope with recovery. For a few, they were in a coma or faced challenges with aphasia (trouble with language), but none of the 9 participants indicated	

		feeling their doctor or other hospital staff sufficiently answered their questions about their stroke. (10)<br><br>**Peers helped stroke survivors in learning more about their conditions and how to cope with their lives.**<br><br>We don't learn enough. Doctors never told me anything." –Lillian, 1.5 years post-stroke<br>(10)<br>"I wanted a place to learn more and educate myself more on stroke and how to cope with life." –Richard, 2 years poststroke (10)<br><br>I didn't understand stroke until I went home and started seeing other stroke survivors." –Tom, 7 years post-stroke (10)<br><br>**The video narrative intervention significantly increased stroke related knowledge.**<br><br>The Stroke Knowledge Test (SKT) substantial mean score differences between groups (F (1214) =11.54, p=0.001, η2=0.051)<br><br>(F = 11.54, $p < 0.001$) (14)<br><br>**The group enhanced knowledge about stroke support services in the community and opportunities such as voluntary jobs and modified leisure activities.**<br><br>Finally, members identified on the survey that the most useful aspect of the group process was learning about new **information, education, and information** on community re- sources. Thus, results from the survey supported both social engagement and role participation. (5)-	

TABLE 5. Member Responses to Post participation Survey ($N = 13$) (5)

Answer Options	Strongly Agree	Agree	Neutral	Disagree	Strongly Disagree
I have made new friends	5	5	2	0	1
I provided support to other group members	5	4	3	0	1
I learned new ways to cope with issues related to stroke	3	7	2	0	1
I am using a coping strategy learned in the group to deal with frustrating or stressful situations	2	6	4	0	1
**I know more about community and stroke resources**	**5**	**5**	3	0	0
I have gained knowledge to advocate for myself	3	7	3	0	0
I feel more hopeful about the future	4	5	4	0	0
**I know more about opportunities after stroke (i.e.,** modified leisure, volunteer, or research opportunities)	**4**	**6**	2	0	1
I am more informed about ways to achieve my goals regarding recovery	4	7	2	0	0

Note:1= Kessler *et al.,* (2014);2=-Morris and Morris (2012);3= Clark *et al.,(* 2018);4=-Masterson-Algar *et al.,* (2018);5=Muller *et al.,(* 2014);6=Chow (2018);7= Christensen *et al.,(* 2019);8=- Gurr (2009);9= Kirkevold *et al.*, (2014);10= Hancock, (2009);11= Corsten *et al.*, (2014);12= Corsten *et al.*, (2015);13= Appalasamy *et al.*, (2020a); 14= Appalasamy *et al.*, (2020b)

Table S9 the second stage of the thematic development and CerQul Assessment

Theme	Subtheme	Code	CerQual Assessment
**Outcomes from the intervention**	**Introducing the concept of hope and learning to be positive regarding the future after stroke**	**Definition of subthemes: discussion around how the intervention of the interest in both individualised and group formats, tended to aid in the introduction of the concept of hope following a stroke diagnosis and in assisting stroke survivors to be optimistic about the future which occurred through the following mechanism.**<br><br>1. Talking to someone who had gone through a similar situation and taking time to listen and shar stories of others was identified as	Methodological limitations: minor concerns.<br>Coherence (fit between studies and review findings: No concerns<br>Adequacy of data (Richness): No Concerns<br>Relevance (applicability to context): No Concerns<br><br>Confidence in evidence rating: High confidence

		validating the feeling of suffering (affirmation support) (1)<br>2. Being in a group with other stroke survivors who were in a similar situation and observing how they improved helped participant to learn to be positive regarding the future (2).<br>3. The narrative aspect of the intervention (Listening to the recovery stories of other stroke survivors could led to the realisation that a person after stroke could recover which as consequence installed hope that did not exist previously (10).<br>4. Being in a group with people with similar situation could led to unite the experiences (validate the suffering) (10)<br>5. Stroke survivors were able to share how they effectively handled challenging circumstances following their stroke in a group setting, which helped others to learn how to handle their own situations. (2). (10) and (9) and were able to apply what they had learned in the group to the management of their lives (2)<br>6. Humour and positivity in groups were also frequently mentioned as important components of effective recovery (10).	

		that emotional support was provided during the initial in- hospital visit in the form of hope, encouragement and re-assurance .Emotional and affirmational support occurred through taking time to listen, sharing of stories and validation of feelings(1)<br><br>:I honestly didn't want them to leave because I just wanted to continue talking to someone who actually had gone through what I went through. (New Stroke Survivor-Sylvie, initial interview) (1)<br><br>I've learnt to stay positive by seeing people who were in similar situations who are now a lot better... being with another who had had a stroke was encouraging as she was in my situation... I feel more positive for the future..... If I hadn't have attended the group I wouldn't be as positive as I am now." [Patient 7]) (2)<br><br>Eleven participants, 5 recipients and six peer support-, agreed with the item "things seem more hopeful since joining the group (2)<br><br>seventeen participants, all except one of the peer supporters, agreed with the item " group helps me feel more positive about my future". (2)<br><br>Sixteen participants, all except one recipient and one carer, agreed with the item " is group inspires me about the future". (2)	

		Just by going and listening to their [other survivors] stories…there is really something to that. Learning the stories of recovery … helped me [learn] people can recover. It gave me hope where I thought through the years that you couldn't recover." –Irene, 5 years post-stroke (10)<br><br>We're not alone. Everybody else is the same as us. I've learned from coming [to the group] that there is life after stroke." –Tom, 7 years post-stroke (10)<br><br>There may be limits to the physical comeback that you can make but as far as keeping yourself positive, which is a constant battle … this group and other avenues too: church, your other relationships with people …That's the biggest thing on keeping your mental positive attitude going, and in the final analysis that's almost everything." (7)<br><br>Fourteen participants, eight recipients and six peer 2 supporters, agreed with the item "When I hear how other people are coping with their problems in the group, I often **learn new ways** to cope with my difficulties".(2)<br><br>13 participants, eight recipients and five peer supporters, agreed with the item "I pay attention to how others handle difficult situations in my group so I **can apply these strategies in my own life (2)**<br><br>The participants in the group-based intervention also reported that **by listening to how oth**er stroke survivors managed their situation, they **learned new** ways to	

		approach different situations: P: I always left [the meetings] **a little inspired!** I think it is important when a serious thing like a stroke happens, that one may exchange experiences with others who have been in the same situation… . That is what has been most important for me – to be together with people in the same situation. (9)<br><br>"When they [other survivors] talked about their experiences and how they coped…**I learned** tips on how to get on with it." –Lena, 2.5 years post-stroke (10)<br><br>Laughter and being positive were also commonly raised as key components of a successful recovery and of strong peer support groups (10)<br><br>Viewing the video narratives provided some hope and less fear to overcome stroke challenges.<br><br>The video was an aid to their plight that there **was life after stroke.(13)**<br><br>They had a **positive** outlook towards stroke recovery and were willing to do better to improve their health condition.(13)<br><br>I feel that I have to follow the advice, for example, taking medicine, doing blood test… that have been mentioned… (The videos) seem to **inspire** us to take care of health so that we won't get sick. Perhaps to give encouragement makes me feel that I can recover from stroke if follow all the advice [P5] Usually, if you never had a stroke before, you don't really care about watching the videos. Once you had (a stroke), you'll realize that… health is	

		important… you have to take care of it… watch their story… that's it! [P1] Now I ask my doctor more questions if I don't understand…. [P2](13)<br><br>The noted change patterns over time were further investigated in their mean plots, which suggested that the patterns of change over time in the outcome measures across the intervention and the TAU groups were statistically different. Further, many of these positive effects were sustained at 4 months post intervention, in the intervention. Pairwise comparisons revealed that hope, meaning in life, self-esteem and stroke knowledge had significant changes between T 0 and T 1 , T 0 and T2, and T 0 and T 3 (Table 3) (6)<br><br>**Intervention group**<br><br>T0-T1: p = .01**<br><br>T0-T2:p <.001*** (6)<br><br>T0-T3p =.04*<br><br>**Control group:**<br><br>T0-T1 :ns<br><br>T0-T2 p =.06	

		T0-T3: ns (6)<br><br>NT shows a higher improvement in most outcome scores over the conventional psycho-education intervention. Results further indicate that the intervention group significantly improved hope ($R2=0.04$, $F (1, 176)=7.24$, **$p =$ .05), (6)**<br><br>**The item of the helpfulness of group " share your story" on a 10-point Likert-type rating was completed by nearly half of the participants (n=37/80) and the result showed an average rating being 7/10.**<br><br>The Likert-rating item on helpfulness of the group for the participants' recovery was completed by 37 participants. The ratings of helpfulness of the group showed an average of 7 (range 1–10).(8)	
	**The enhanced the ability to cope**	**Definition : discussion around how the group context enhanced ability to cope and recovery after stroke which occurred through the following mechanisms :**<br>**(1) Meeting other stroke survivors who had positive experiences of recovery was motivating to new stroke survivors (1)**<br>(2) Increasing knowledge about stoke not only helped stroke survivors feel more educated but also gave the skills that they could use outside	Methodological limitations: minor concerns.<br>Coherence (fit between studies and review findings: moderate concerns<br>Adequacy of data (Richness): moderate concerns<br>Relevance (applicability to context): moderate concerns

		the group context to facilitate recovery process (10) and (7)<br>(3) This could be aided by a source of self-efficacy. There was a significant increase in self efficacy, when compared to a control group, the intervention group had a higher level of self-efficacy (F = 12.41, $p < 0.001$) see **Table 2** Groups' Comparison at Various Timelines (14)<br><br>The visit from the peer supporters encouraged and motivated the stroke survivors to work towards recovery (1)<br><br>Peer support groups helped survivors understand their stroke (10)<br><br>Many participants indicated that at the time of their stroke and during their hospital stay, they did not feel they adequately understood their stroke **or how they could cope with recovery.** For a few, they were in a coma or faced challenges with aphasia (trouble with language), but none of the 9 participants indicated feeling their doctor or other hospital staff sufficiently answered their questions about their stroke. (10)<br><br>Having stroke-related knowledge not only made participants feel more informed, but also equipped them with concrete skills they applied at home to further their recovery (7)	Confidence in evidence rating: low confidence

		Ten of the 13 members reported "agree" or "strongly agree" they were able to identify a new coping strategy, and eight of the 13 members reported they actively used the strategy outside the group. (5)<br><br>These findings provide preliminary indication that the YESS group had a positive impact on coping strategies after stroke. (5)<br><br>For example, members were asked if they engaged in volunteer activities, exercise programs, or leisure programs that were presented in the group modules. Over half of the members (n = 8) reported they began to engage in various leisure and ADL opportunities outside of the group context. (5)<br><br>**Being supported in their attempts to cope with the situation (9)**<br><br>The participants struggled to cope with their new and unknown situations after the stroke. The issues they struggled with varied widely, **from performing daily activities and solving practical problems to understanding and coming to terms with their own emotional reactions and those of their family, friends and colleagues**. Facing different social situations within and beyond their family entailed many challenges. (9)<br><br>The participants reported that the intervention helped them cope with their struggles. Participants in both the individual and group-based interventions emphasised that the dialogues helped them cope by **clarifying what their coping challenges entailed, illuminating their**	

		**coping options, supporting them as they tried different oping strategies and support**ing them as they analysed unexpected situations (9)<br><br>fifteen of the participants, eight recipients and 7 peer supporters, also agreed with the statement "In the group, other members advise me what I should do about difficult situations and life decisions(2)<br><br>Nine participants, four recipients and 5 peer supporters, agreed with the statement "In the group I get 'how to on improving my life (2)<br><br>**Main theme: Informative and reminder • The videos narratives were a "trigger" toward proactivity and enhanced patients' awareness about stroke and its preventative treatment**. (13)<br>They remind us of important medicine… They remind us of the danger of the second stroke… to take medicine well and to have a healthy lifestyle [P7] Helpful….more understanding about stroke [P6] (13)<br>Awareness… before that we were not really concerned about our health. Now, after the advice it's different... like a guide [P4] (13)<br>Patients can recover from stroke and (it) won't recur if we take the medicine prescribed by doctors according to the right schedule on time [P2] (13)<br><br>For example, members were asked if they engaged in volunteer activities, exercise programs, or leisure programs that were presented in the group modules. **Over half of the members (n = 8)** reported they began to engage	

		in various leisure and ADL opportunities outside of the group context. (5)<br><br>**Handicap domain of Stroke Impact Scale (SIS)** *p* value= .034<br>**Home integration domain of Community Integration Questionnaire (CIQ)** *p* value= .002 (5)<br><br>**the SIS self-perceived recovery domain and the social and productivity integration domains of the CIQ did not reach significance**<br><br>**the SIS self-perceived recovery =*p value*=.196**<br><br>**productivity integration domains of the CIQ**<br>*p value* =0.334	
	**Impact on loneliness and social interaction**	**Definition: discussion around how the intervention of interest had positive outcomes on social well-being which were the following: (1) decreasing the sense of loneliness following stroke,**<br>**<u>Related to decreased loneliness.</u>**<br><br>**<u>Related to decreased loneliness.</u>**<br><br>After having someone who had gone through a similar experience take time, listen, share experiences, and make a connection, stroke survivors reported feeling validated and less alone(1)<br><br>It made me realise **I was not alone**, that there are many others in the same position." [Patient peer supporter 2]. (2)	Methodological limitations: minor concerns.<br>Coherence (fit between studies and review findings: No concerns<br>Adequacy of data (Richness): No Concerns<br>Relevance (applicability to context): No Concerns<br><br>Confidence in evidence rating: High confidence

		shared experience may **reduce feelings of loneliness as Paul illustrates** (3)<br><br>That is good if you know there are others in the same position as you. Going, going through the same thing as you. So you don't think you are the only one... It's good talking to other stroke survivors because with their experiences you know you are not alone then. Because it is very demoralising once you have had your stroke. (Paul) (3)<br><br>-I think partly it was like a day out for her, breaking the monotony, but once her family came back she had something to fill the day. When I had my stroke and was looking for help, anything I was offered I would go for it. For me I was looking for solutions to problems, I was meeting with her to work on solving problems. Her problem **was loneliness, so I was the solution**. (Peer 2) (4)<br><br>Participant R2G3 said, "It makes you feel like you **are not alone.** Like there is someone else out there that is having the same problems and concerns that you do. And how they are dealing with it makes a difference(7)<br><br>-I think that if I had' t had this course [intervention], I would have felt terribly alone. (Woman, 66 y.o./**group intervention).(**9)	

		Peer support groups helped survivors not feel alone or isolated. **Almost all participants commented on the importance of connecting with other survivors**. **At least half of the survivors expressed gratitude to ha**ve received either a visit in the hospital or a phone call from a stroke survivor. They **articulated that the isolation of their stroke seemed somewhat alleviated once they connected with others in a similar situation**. “You find you’re not alone. [The group] is an extended family who knows what you’re going through.” –Angela, 11 years post-stroke (10)<br><br>You don’t feel quite so much out on a limb. When you’ve heard [that] other people have gone through the same procedures you had.” **[Patient 6].** (2)<br><br>“It made me realise I was not alone, that there are many others in the same position.” [**Patient peer supporter 2].** (2)<br><br>**<u>Related to developing valuable relationships and increased social interaction which occurred through the following mechanism:</u>**<br><br>it the group has changed my life **as I have made friends with other people who** have had a stroke.” [Patient 7].(2)<br><br>Member response to post participations survey :5 out 13 reported “strongly agree “ that the program helped them to make new friends and 5 out of 13 reported” agree” (5)	

		Descriptive data about social and communication activity outside of the group were tracked on the post group survey. For example, members were asked if they met with anyone socially outside of the group or if they used communication path- ways such as Facebook or texting to socialize/communicate outside of the group. Approximately, half of the individuals (*n* = 6) reported interaction with another member outside of the group context in various formats including text (*n* = 2), Facebook (*n* = 2), met or attempted to meet outside of the group (*n* = 8), email (*n* = 5), and talk on the phone (*n* = 6). (5)<br><br>Equally, over half of the members (***n* = 8) reported socialization being their favourite aspects of the** group process .example, members reported new friendships and social networking were most beneficial. (5)<br><br>The social domain<br><br>The CIQ social domain did not reach significant:<br>Mean pre score: 8.23<br>Mean post score: 9.08 (5)<br>***p* value =0.148) (5)**<br><br>The sense of community was unique to the support group experience, and impacted participants' lives beyond scheduled stroke support group meetings. Participant R3G2 noted, "I think this group has become more than a support group once per month. They are almost like family (7)<br><br>They [other survivors] speak to me and then they'll listen. Some people would say 'oh, I can't be bothered'. **They're like friends** because they're always willing to help you'" –Lillian, 1.5 years post stroke (10)<br><br>**Negative case :**	

		The peers identified that although they had not experienced this difficulty, they could see how there was the potential for the stroke survivor **to become attached to the peer: I**t's hard to meet somebody one to one. To meet them at a personal level and then suddenly, that is it, you are never going to see them again. This could be a problem because if you get the wrong sort of people, the ones that cling on all the time they would be phoning you all the time just for a bit of company. (Peer 1) (4)<br><br>**Proposed mechanism behind evidence :**<br>(1) Exchanging stories with other stroke survivors who had gone through similar circumstances was identified as essential inspiring and necessary, particularly when dealing with something as severe as the occurrence of stroke.<br>(2) Peers were also seen as a source of inspiration to pursue a peer position in the future, which aided in the growth of **social identity.**<br>(3) The facilitators gained a better understanding of the participants as individuals because of sharing stories with the groups (8)<br><br>(4) Sense of community that was created in the group could led to develop a lifelong valuable relationships , support and network outside of the group context that continued validating the sense suffering outside the group context .(7)	

		When a traumatic event occurs, such as a stroke , **it is important to be able to share experiences with those** that had been in similar situations (9)<br><br>Peer supporters were also seen as a source of inspiration (1)<br><br>some stroke survivors who expressed an interest in pursuing a peer supporter role in the future.(1)<br><br>As part of the evaluation, the facilitators were asked to answer the following question by the trainee clinical psychologist: ‘How was the Share **Your Story Group useful for you as therapi**st?’ Responses were written down verbatim and grouped. This resulted in the following categories: (8)<br><br>■ Getting to know patients as people (six responses (8)<br><br>**They have become friends and support** outside of the meetings.” Additionally, participant R2G1 stated, “We love the relationships that we are building with other people. Even if we are in a store ... it’s like, ‘oh they are in our stroke group.’ It is important that outside of this room even, it reminds you that there are other people experiencing the same reconfiguration of their life that we are.” (7)	
	**Impact on emotions**<br><br>**Depression**	**Definition: discussion around the impact of the intervention of interest on depression and related emotions**	Methodological limitations: minor concerns.

		**Qualitative results related to depression outcome :**<br><br>**Mechanism related to depression outcomes** :<br><br>**1.** sharing experience with other stroke survivors and validating own suffering helped in reducing depression. (7) (10)<br><br>Many participants shared feelings of frustration, depression, and isolation. These feelings stemmed from fear, lack of strength, and feeling overwhelmed by the challenges of adjusting to a new normal after stroke. However, **sharing their experiences in peer** stroke support groups was helpful. Participant R2G1 noted, "I felt that I was a **little depressed** before I came … I found that being around **other people that's gone through the same thing helps."(7)**<br><br>2. Being in a group of people with similar condition and who had disabilities ,yet had positive attitudes could led to increase spirit and decreased depression (10)<br><br>3. Participating in a peer support group provided patients with something to do once a month, which helped to alleviate depression. (10)<br><br>Although many survivors experience depression post-stroke, their support group played a role in overcoming depression (10)	Coherence (fit between studies and review findings: moderate concerns<br>Adequacy of data (Richness): moderate concerns<br>Relevance (applicability to context): moderate concerns<br><br>Confidence in evidence rating: low confidence

		Of those who experienced stroke, the majority expressed their support group played a significant role in overcoming depression. (10)<br><br>“Being together with such a happy group who had disabilities too helped me keep my spirits up so I don’t let myself get down.” –Lena, 2.5 years post-stroke (10)<br><br>“I personally thought about suicide. It’s scary. In forums they [survivors] all said we felt depressed…in the beginning it’s very normal. I’m not alone feeling that way.” – Angela, 11 years post-stroke (10)<br><br>They [the support group] helped a lot. I talked about depression and why I wanted to commit suicide and they were really supportive.” –Tom, 7 years post-stroke (10)<br>When I first came home from the hospital I wanted to go home, sit in a corner and cry…..now every month I have something to go to.” –Aryln, 7 years post-stroke (10)<br><br>**Quantitative results regarding depression :**<br><br>Intervention group:<br>T0(baseline),*M(*SD).32(.25);T1(interim) .33(.27);T2(post intervention),-.25(.26) and T3(4 months)-.20(.26) (6)<br><br>A reduction in the mean, for both anxiety and depression scores was found.<br>Mean pre group depression approximate just **at 8.5 of** Hospital Anxiety and Depression Scale (HADS) and	

Mean of post group depression of HADS approximately just **above 7.5** (8)

, but T-test results showed no significant changes in HADS scores before and after the group (n=34) (8)

Affective mood states, measured by the VAMS at the end of the first and the last BNI also changed, as shown in table 4. While **little improvement was demonstrated for the mood state 'happy'** with an increase in the mean T-score from 40.85 to 47.40 (Cohen's d = .25), the mood states 'confused' and 'tense' decreased moderately from 52.96 to 46.23 (Cohen's d = .56) and from 53.52 to 46.50 (Cohen's d = .45), respectively. For all other mood states there were expected trends towards a growth of positive emotions and a reduction of negative emotions. We also conducted **the VAMS at the end of the first and the last GI. Again we found expected trends. The mood state 'tired' decreased from 49.00 to 43.33 (Cohen's d = .30) (12**)

Table 4. Values for mood states VAMS (Stern 1997) at end of the first and the last biographic–narrative interviews (BNI) and group interventions (GI) (12)

Test	Baseline (end BNI 1) (n = 27), mean T-scores (95% CI)b	Post (end BNI 5) (n = 26), mean T-scores (95% CI)	Baseline versus post-test P-value (outcome)a	Cohen's d
VAMS afraid	47.41 (2.61)	46.62 (1.68)	.50	.12
VAMS confused	52.96 (5.77)	46.23 (2.12)	.01	.56
VAMS	52.37	47.81	.08	.30

sad	(6.73)	(3.25)		
VAMS angry	50.04 (5.10)	47.08 (3.39)	.25	.21
VAMS energetic	44.37 (5.11)	48.46 (5.02)	.27	.14
VAMS tired	46.96 (4.00)	46.92 (4.22)	.78	−.03
VAMS happy	40.85 (5.48)	47.40 (4.52)	.01	.25
VAMS tense	53.52 (4.42)	46.50 (2.97)	.00	.45
	**Baseline (end GI 1) (*n* = 25), mean *T*-scores (95% CI)**	**Post (end GI 7) (*n* = 24), mean *T*-scores (95% CI)**		
VAMS afraid	47.33 (4.73)	46.86 (3.27)	.40	.36
VAMS confused	48.00 (5.48)	45.35 (2.03)	**.40**	.58
VAMS sad	52.96 (8.85)	47.76 (5.24)	.32	.38
VAMS angry	48.75 (6.54)	45.19 (2.15)	.37	.41
VAMS energetic	45.25 (6.03)	48.62 (15.17)	**.48**	−.09
**VAMS tired**	**49.00 (6.99)**	**43.33 (11.70)**	**.00**	.30
VAMS happy	42.88 (6.92)	45.52 (14.92)	**.23**	−.02
VAMS tense	51.21 (4.86)	49.86 (11.80)	**.93**	.18

Notes: [a]Repeated measures *t*-test, two-tailed.
[b] *n* varies because not all participants attended at each session.

TABLE 4 Values for mood states VAMS (Stern, 1997) at end of first and last biographic-narrative interview (BNI) and group intervention (GI) (*n* varies because not all participants attended at each session) (11)

	Baseline (end BNI 1) *(n = 17)* *Mean -t-scores (95% CI)*	*Post (end BNI 5);* *(n = 16)* *Mean t-scores (95%CI)*	*Baseline vs.Post-test p-values[1] (outcome)*
**VAMS** afraid	48.06 (4.32)	46.38 (2.35)	.30
VAMS confused	54.88 (8.99)	46.50 (3.65)	.03*
VAMS said	52.88 (10.33)	48.13 (5.33)	.12
VAMS angry	52.71 (8.42)	47.44 (5.49)	.20
VAMS energetic	44.35 (7.33)	49.00 (7.11)	.39
VAMS tired	48.65 (6.12)	46.38 (5.47)	.64
VAMS happy	39.00 (7.66)	46.53 (7.13)	.02*
VAMS tense	54.47 (6.59)	46.63 (4.49)	.01**

Test	*Baseline (end GI 1);* *(n = 14)* *Mean t-scores (95% CI)*	*Post (end GI 7)* *(n = 13)*	*Baseline vs. Post-test* *Mean t-scores (95% CI)* *p-values[1] (outcome)*
**VAMS** afraid	48.00 (4.44)	46.00 (2.70)	.56
VAMS confused	49.43 (5.48)	45.00 (1.53)	**.30**
VAMS said	51.64 (8.85)	46.23	.42

<table>
<tr>
<td></td>
<td></td>
<td>
<table>
<tr><td></td><td></td><td>(3.37)</td><td></td></tr>
<tr><td>VAMS angry</td><td>49.00 (6.54)</td><td>44.69 (1.55)</td><td>1.00</td></tr>
<tr><td>VAMS energetic</td><td>44.93 (6.03)</td><td>47.46 (8.09)</td><td>.86</td></tr>
<tr><td>VAMS tired</td><td>52.07 (6.99)</td><td>45.15 (6.61)</td><td>.06</td></tr>
<tr><td>VAMS happy</td><td>42.36 (6.92)</td><td>43.92 (8.25)</td><td>.46</td></tr>
<tr><td>VAMS tense</td><td>49.79 (4.86)</td><td>46.23 (5.67)</td><td>.44</td></tr>
</table>
[1]Repeated measures t-test, two-tailed.
Definition: discussion around how the intervention of interest seemed to reduce the sense of fear and instilling a feeling of reassurance
Qualitative result :
Viewing the video narratives provided some hope and less fear to overcome stroke challenges. It's a bit of both worrying and confidence… There is always a worry about what can happen, but it also gives you an idea (on) what to do, and what to be careful, and what to be aware [P3] (13).
That emotional support was provided during the initial in-hospital visit in the re- assurance(1)
far was emphasized as a great challenge in accepting stroke..
[10]
When I first had the stroke, I went to my family doctor 3-4 times a week thinking I would be having a stroke. I was always scared," and indicated "the group helped me overcome my fear." –Kelly, 4 years post- (10)
</td>
<td>Methodological limitations: minor concerns.</td>
</tr>
</table>

		that's **where the group helped**." Irene, 5 years post-stroke (10)<br>The motivational cues inspired the patients and raised confidence among themselves. … (sharing) someone's experience to change others 'mind. Sometimes, we need to listen to their stories for us to make a change [P4] (13)<br><br>**Negative case** :There may conversely be some negative impacts of the interventions on emotions of both stroke survivors and peer supporters which might occurred through the following mechanism :<br><br>• Some aspects of the intervention such as sharing experiences of stroke and recalling distressing memories might be "distressing" and "difficult" (3)<br>• Sharing experiences of stroke within a group context might impact negatively on physical and emotions health (increased stress) (3)<br><br>**Negative case related peers** who provided the intervention : there might be a potential emotional impact of the intention on peers as the intervention might trigger experience relates to their initial stage of stroke.<br><br>While concern was expressed by program coordinators about the potential emotional impact on peer supporters for whom the visit may trigger past feelings related to the initial experience of stroke, none of the peer supporters interviewed described experiencing these types of feelings (1)	Coherence (fit between studies and review findings: moderate concerns<br>Adequacy of data (Richness): moderate concerns<br>Relevance (applicability to context): moderate concerns<br><br>Confidence in evidence rating: low confidence

		Ability to cope emotionally – "perhaps not everyone can cope" The ability of each individual to cope with the potential emotional demands of a group SMP was discussed by participants. Some aspects of a group may be emotive for some individual's such as, **discussing experiences of stroke and remembering things that are distressing. (**3)<br><br>Five stroke survivors spoke about how they would cope emotionally when hearing and talking about stroke. Thomas reflects on times he has had to hear about stroke, such as in the paper. He says he does not like it as he finds it both "**difficult**" and "**distressing"** to hear(3)<br><br>**Quantitative result :**<br><br>**VAMS** afraid (P=.056) (11)<br>**VAMS** afraid (p= 0.36) (12)	
	**enhanced awareness of stroke and knowledge**	**Definition: discussion around how the intervention of interest provided information support which enhanced awareness of stroke and increased knowledge**<br><br>**One reason to join the group was pursing and gaining knowledge related to stroke (7) and (10)**<br><br>Theme 1: Survivors and caregivers **seek and receive** knowledge about stroke and stroke recovery from stroke peer support groups. (7)<br><br>Participants shared that **they initially attended** stroke peer support groups to learn more about stroke and	

		recovery (7)<br><br>"I wanted a place to learn more and educate myself more on stroke and how to cope with life." –Richard, 2 years poststroke (10)<br><br>**The intervention of interest provided useful knowledge about stroke prevention and rehabilitation, as well as identifying and accessing stroke support services in the community, to stroke survivors and their care partners.**<br><br>Informational support was also offered during the initial and follower up telephone visits. Peer supporters provided written as well as verbal information on resources in the community including those offered by SSAO. (1)<br><br>During the initial visit this information tended to be more general in nature. However, during follow-up telephone calls, information was targeted to the new stroke survivor's or care partner's needs and included information on specific services avail- able in the community. Information to assist with both finding and accessing services was provided to six out of the 28 people for whom follow-up diaries were kept (1).<br><br>What did these **survivors want new** survivors to know about stroke peer support groups? (10)<br><br>"They can help you and be very helpful with resources." – Kelly, 4 years post stroke (10)	

		Importantly by meeting with other stroke survivors at different stages of recovery, participants gained knowledge from each other and felt empowered by providing help to their peers. Participant R2G4 said, "Each one of us helped somebody that was having a stroke. If we can do what we do and not be doctors, we can make a big difference right here." (7)<br><br>You discuss things so you can be on the lookout for anything that might be going wrong, or things you need to be looking to get checked out if you are having this symptom or that one that you might not have thought about before(7)<br><br>Several participants felt that peers provided valuable information and advice about post-hospital support services (2)<br><br>I've learnt what help you can get when you come home... and how to get it." [Patient 5].(2)<br><br>**Information about the experiences of coping with stroke provided by peers was valued higher than the information provided by health care professionals.**<br><br>Information received from a peer regarding the experience of living with stroke was generally given more value than that received from a healthcare professional.(1)<br>And (10)	

		**Stroke survivors who had a lot of experiences since their stroke thought to be the in the best position to support.**<br><br>Individuals with substantial experience subsequent to their stroke were viewed as being in the best position to be helpful. (2)<br><br>"that was the ones that I probably got the most benefit from. the ones that had come in that had had strokes probably a year earlier than I had that were further up the road...and you can ask them questions... what do you do in this situation or another situation?" [Patient 6]. (2)<br><br>**Peers offered knowledge and information gained from real -life experiences which promoted relatedness.**<br><br>peer supporters] provided sort of reassurance... and provided a real face. When you're dealing with doctors and nurses they're great.... But they're medical people and they can talk to you about what you've just gone through, but the chances are more than likely that they haven't... The people in Stroke Survivors, well they have. (New Stroke Survivor-Mike, initial interview) (1)<br><br>**The group enhanced knowledge about stroke support services in the community and opportunities such as voluntary jobs and modified leisure activities.**	

		Finally, members identified on the survey that the most useful aspect of the group process was learning about new **information, education, and information** on community re- sources. Thus, results from the survey supported both social engagement and role participation. (5)-<br><br>See TABLE 5. Member Responses to Post participation Survey (*N* = 13)<br><br>**It was difficult to obtain knowledge about stroke and rehabilitation from stroke, and they and their caregivers valued the ability to learn about these topics from both peers and experts**<br><br>Receiving stroke and recovery related knowledge was important to survivors and caregivers, and difficult to locate. As participant R2G1 stated, "We all go different places and do different things but there is not really a great one source; you just can't go to the kiosk and say, 'I would like to have all of these resources for stroke survivors.' It's just not there." (7)<br><br>**The video narrative intervention significantly increased stroke related knowledge.**<br><br>The Stroke Knowledge Test (SKT) substantial mean score differences between groups (F (1214) =11.54, p=0.001, η2=0.051)<br>**(F = 11.54, $p < 0.001$)** (14)	

Note:1= Kessler *et al.,* (2014);2=-Morris and Morris (2012);3= Clark *et al.,(* 2018);4=-Masterson-Algar *et al.,* (2018);5=Muller *et al.,(* 2014);6=Chow (2018);7= Christensen *et al.,(* 2019);8=- Gurr (2009);9= Kirkevold *et al.*, (2014);10= Hancock, (2009);11= Corsten *et al.*, (2014);12= Corsten *et al.*, (2015);13= Appalasamy *et al.*, (2020a); 14= Appalasamy *et al.*, (2020b)

S10: The details of the intervention included.

Elements of each intervention can be seen in Table S2. Various types of intervention were identified; Seven [32,34-36,39-41] included a narrative based intervention. Five other studies took a peer sharing based approach in a one-to-one format [31,37] or within groups [29,30,33] and the two remaining studies used peer support in self-managed intervention groups [28,38].

The interventions were hospital based in five studies [33,35,36,38, 39]; six were based in the community [29,30,34,37, 40, 41]; two [31,32] combined hospital and community; one [28] was unknown. Two interventions were peer led [31,37], five re professionally led [32,34,36,40,41] , two were video narrative presentation [35,39] and the rest were a combination of peer and professionally led [29;38] or were led by a combination of peers, partners of care or family members and healthcare professionals [30,33 The details of the intervention are included] and one was unknown [28]. The duration and the frequency of intervention sessions varied from 10 minutes to two hours, repeated weekly or monthly for two weeks to 12 years.

Frequency of sessions:

Three of the fourteen studies reported on the frequency and duration of the intervention and the number of sessions [31,32,37]. Peer supporters suggested monthly meetings were too little [31]. Participants who had a Stroke recommended weekly meetings, with frequent meetings at the beginning being the most important [32]. Both peer supporters and stroke patients emphasised the importance of having a fixed number of sessions, as it helped in planning and defining what the process of the intervention would be and then in keeping to the plan, not going too quickly or too slowly [37].

Duration of sessions:

The individualised sessions were planned to last one hour and the group sessions two hours. However, while most of the individualised sessions, especially for those without language difficulties, were completed within an hour, the duration of sessions differed among those with language difficulties, especially in the early stages of the stroke journey, as they became quickly tired in their attempts to express themselves. Therefore, the sessions needed to be individually modified and lasted on average one and half hours [32].

Tailored intervention format:

Only two studies [31,37] provided the intervention in an individualised format and the opinions of participants were generally positive regarding the format of the intervention they engaged in. In the study by Kessler et al. [31], new stroke patients and health professionals reported that individualised encounters with one peer supporter were preferable and that receiving a visit from two peer supporters was acceptable, whereas more than two could be overwhelming to new survivors. Although Masterson-Algar et al. [37] did not directly evaluate the format of the intervention, both stroke survivors and peer supporters reported having positive outcomes at personal level after engaging in one-to-one peer-led coaching sessions.

On the other hand, nine stroke participants (n=9/14) in the study by Clark et al. [28] appreciated the potential value of both emotional and practical support provided by peers within the group, in particular the potential benefits of shared problem solving. One participant explicitly reported that she preferred a group format over one-to-one, which she felt might be restricted to exposing her to the ideas of only one person, whereas a group of peers would offer a wider and more varied perspective and set of ideas on how to manage difficult situations [28]. Stroke survivors who received the group dialogue format and the individualised dialogue with an HCP suggested that the group format provided a platform for sharing experiences and exchanging ideas, but it was more difficult to address individualised needs than in the individualised format; thus, some participants found the group format to be limiting to some extent, as the intervention concentrated on topics and concerns common to many participants and less on individualised concerns [32].

Only one study discussed group size and reported mixed views about the optimal size [33]. Some participants reported anxiety about sharing when groups were larger than five or six members, while some partners of care suggested that smaller groups could motivate more people to engage in the discussion.
